# No one tool to rule them all: prokaryotic gene prediction tool annotations are highly dependent on the organism of study

**DOI:** 10.1093/bioinformatics/btab827

**Published:** 2021-12-07

**Authors:** Nicholas J Dimonaco, Wayne Aubrey, Kim Kenobi, Amanda Clare, Christopher J Creevey

**Affiliations:** Institute of Biological, Environmental and Rural Sciences, Aberystwyth University, Aberystwyth SY23 3PD, UK; Department of Computer Science, Aberystwyth University, Aberystwyth SY23 3DB, UK; Department of Mathematics, Aberystwyth University, Aberystwyth SY23 3BZ, UK; Department of Computer Science, Aberystwyth University, Aberystwyth SY23 3DB, UK; School of Biological Sciences, Queen’s University Belfast, Belfast BT7 1NN, UK

## Abstract

**Motivation:**

The biases in CoDing Sequence (CDS) prediction tools, which have been based on historic genomic annotations from model organisms, impact our understanding of novel genomes and metagenomes. This hinders the discovery of new genomic information as it results in predictions being biased towards existing knowledge. To date, users have lacked a systematic and replicable approach to identify the strengths and weaknesses of any CDS prediction tool and allow them to choose the right tool for their analysis.

**Results:**

We present an evaluation framework (ORForise) based on a comprehensive set of 12 primary and 60 secondary metrics that facilitate the assessment of the performance of CDS prediction tools. This makes it possible to identify which performs better for specific use-cases. We use this to assess 15 *ab initio*- and model-based tools representing those most widely used (historically and currently) to generate the knowledge in genomic databases. We find that the performance of any tool is dependent on the genome being analysed, and no individual tool ranked as the most accurate across all genomes or metrics analysed. Even the top-ranked tools produced conflicting gene collections, which could not be resolved by aggregation. The ORForise evaluation framework provides users with a replicable, data-led approach to make informed tool choices for novel genome annotations and for refining historical annotations.

**Availability and implementation:**

Code and datasets for reproduction and customisation are available at https://github.com/NickJD/ORForise.

**Supplementary information:**

Supplementary data are available at *Bioinformatics* online.

## 1 Introduction

Whole genome sequencing, assembly and annotation is now widely conducted, due predominantly to the increase in affordability, automation and throughput of new technologies (Land *et al.*, 2015). The prediction of protein-coding genes, specifically their corresponding CoDing Sequence (CDS) in prokaryote genomes has often been seen as an established routine. This is in part due to a number of assumptions and features, such as the high density (protein-coding genes contribute ∼80–90% of prokaryote DNA) and the lack of introns (Lobb *et al.*, 2020; Salzberg, 2019). However, this process involves the complex identification of a number of specific elements, such as: promoter regions (Browning and Busby, 2004), the Shine–Dalgarno (Dalgarno and Shine, 1973) ribosomal binding site and operons (Dandekar *et al.*, 1998), which all contribute to identifying gene position and order. Additionally, the role of horizontal gene transfer (Jain *et al.*, 1999) and pangenomes further complicates an already difficult process and likely contributes to errors and a lack of data held in public databases (Devos and Valencia, 2001; Furnham *et al.*, 2012). Finally, our ability to characterize the functions of regions of DNA [which has been generally reserved for model organisms (MOs) and core genes (Russell *et al.*, 2017)] is being outstripped by the rate of genomic and metagenomic sequence data generation from non-MOs and non-core gene DNA sequences.

Before the turn of the century, it was understood that a great deal of work was still needed to address these issues. Studies had shown that many existing CDS prediction tools systematically failed to identify or accurately report genes whose features lay outside a rigid set of rules, such as non-standard codon usage, those which overlap other genes or those below a specified length (Burge and Karlinb, 1998; Guigo, 1997). Since then, a systematic overview of 1474 prokaryotic genome annotations in GenBank concluded ‘the cause of the high rates of missed genes is less clear, largely due to a lack of information about the annotation methods used’ (Wood *et al.*, 2012). Interestingly, while the majority of missed genes reported were under 300 nt, the annotation tools, which performed the incomplete annotations were developed to report CDSs at a minimum length of 110 nt. While there has been much work to address the problem of incomplete annotation, many gene types continue to be absent or under-represented in public databases (Huvet and Stumpf, 2014; Warren *et al.*, 2010), such as short/small-ORFs (short ORFs) (Duval and Cossart, 2017; Storz *et al.*, 2014; Su *et al.*, 2013). This means that CDS prediction methodologies that use information from existing sequences are in turn ill-equipped to identify genes belonging to these underrepresented/missing gene types. It is therefore of paramount importance that we understand the limits of current CDS predictors as our reliance on automated genome annotation of novel genomes continues to increase (Brenner, 1999). Measures to compare both novel and contemporary CDS prediction tools are not well established or universally employed and novel tool descriptions tend to focus on algorithmic improvements rather than carrying out a systematic assessment of where the strengths or weaknesses in their approaches lie. This prevents researchers from gaining meaningful insight into the specific features of genes, which led to them being missed or partially detected, resulting in a lost opportunity to improve our understanding of prokaryote genome content.

Genome annotation is challenging and is not a single step process. CDS prediction, often the first step, is fast, with little user input, but may require augmentation by different methods to supplement the initial predictions. One example is a tool, such as smORFer (Bartholomäus *et al.*, 2021), that specializes in finding short ORFs through the use of RNA-seq, which can detect transcription events under certain environmental conditions. Further examples use sequence conservation scores and homology searches that can use existing database knowledge (Badger and Olsen, 1999; Dunne and Kelly, 2017; ÓhÉigeartaigh *et al.*, 2014). Furthermore, pipelines are constructed [such as PROKKA (Seemann, 2014) and NCBI’s PGAP (Tatusova *et al.*, 2016)] to automate these further rounds of annotation. However, the underlying CDS prediction tools are still core components of these pipelines and are still widely used as standalone tools.

Previous studies, which have evaluated prokaryotic CDS predictors generally only compared a small number of tools, focussing on algorithm design, and did not go into depth when reporting prediction accuracy with few other informative metrics used (Al-Turaiki *et al.*, 2011; Mathé *et al.*, 2002). A more recent study, BEACON (Kalkatawi *et al.*, 2015), considered a small range of metrics including genes ‘denoted as identical, similar, unique with overlap or unique without overlap’ to either a reference annotation or from the output of three pipelines (PGAP, AAMG and RAST). Unfortunately, the types of genes missed were also not investigated further, leading to a lack of understanding of not only why and how they were missed, but also the impact on our biological understanding of the genome as a whole.

Many prediction methods used today are iterations of original concepts and thus are as in flux as the genomic databases themselves. Future development of CDS prediction techniques is now harnessing the recent advances in machine learning and other computational methods. While previous methods involve the construction of models built from organism-specific parameters, such as codon usage, guanine-cytosine (GC), complex motifs and average CDS length (Besemer and Borodovsky, 1999; Stanke and Morgenstern, 2005), opinions are shifting on the use and importance of MOs (Hunter, 2008; Levy and Currie, 2015; Russell *et al.*, 2017). The volume of prokaryotic protein-coding gene sequences have enabled advanced machine-learning approaches, such as neural networks to predict CDSs that share common characteristics with a selection of previously annotated genes. One such example, Balrog (Sommer and Salzberg, 2021) predicts protein-coding genes by training from an array of non-hypothetical protein-CDSs from thousands of bacterial prokaryote genomes and aims to provide gene prediction across diverse species. Machine-learning models can be poor at making predictions for classes (e.g. genes) whose training data exhibit high levels of bias, error, are under-represented for specific groups (e.g. gene families) and groups for which they have not been trained (Schafer and Graham, 2002). In addition to this, prokaryotic gene families are chronically under-sampled (Warren *et al.*, 2010). It is becoming clear that even with these advances in computational approaches, it is unlikely that we will ever be capable of identifying the complete picture of CDS gene diversity without exhaustive experimental work.

To address these concerns, we extensively evaluate a collection of 15 widely used CDS prediction tools that form the basis of most of the annotations deposited in public databases and therefore have largely been used to build the genomic knowledge used by the scientific community. We provide a comparison platform developed to allow researchers to compare 12 primary and a further 60 secondary metrics to systematically compare the predictions from these tools and study the effect on the resulting genome annotations for their species of interest. This allows for in-depth and reproducible analyses of aspects of gene prediction that are often not investigated and allows researchers to understand the impact of tool choice on the resulting prokaryotic gene collection.

## 2 Materials and methods

### 2.1 Current Ensembl genome annotations

Six bacterial MOs and their canonical annotations were downloaded from Ensembl Bacteria (Howe *et al.*, 2020) (available at https://github.com/NickJD/ORForise/tree/master/Genomes). *Bacillus subtilis* BEST7003 strain (assembly ASM52304v1), *Caulobacter crescentus* CB15 strain (assembly ASM690v1), *Escherichia coli* K-12 ER3413 strain (assembly ASM80076v1), *Mycoplasma genitalium* G37 strain (assembly ASM2732v1), *Pseudomonas fluorescens* UK4 strain (assembly ASM73042v1) and *Staphylococcus aureus* 502A strain (assembly ASM59796v1) were chosen for their scientific importance, range of genome size, GC content, assumed near complete and high quality genome assembly and annotation provided by Ensembl Bacteria. They are presented in Table  1 and further information regarding these MOs can be found in Supplementary Section S1.

**Table 1.** An overview of genome composition for the six MOs selected to evaluate CDS prediction tools compiled from data held by Ensembl bacteria

Model organism [assembly]	Genome size (Mbp)	Genes [CDSs]	Genome density [CDSs]	GC content (%)
*B.subtilis* BEST7003 **[ASM52304v1]**	4.04	4133 **[4011]**	88.91% **[87.60%]**	43.89
*C.crescentus* CB15 **[ASM690v1]**	4.02	3875 **[3737]**	90.60% **[90.23%]**	67.21
*E.coli* ER3413 **[ASM80076v1]**	4.56	4257 **[4052]**	86.28% **[84.35%]**	50.80
*M.genitalium* G37 **[ASM2732v1]**	0.58	559 **[476]**	92.03% **[90.62%]**	31.69
*P.fluorescens* UK4 **[ASM73042v1]**	6.06	5266 **[5178]**	84.75% **[84.20%]**	60.13
*S.aureus* 502A **[ASM59796v1]**	2.76	2556 **[2478]**	83.93% **[82.76%]**	32.92

*Note*: Data are presented for all genes and CDS genes in bold square brackets. Note the relatively broad differences in genome size, gene density (percentage covered with annotation) and GC content.

For each of the chosen MOs, two data files were downloaded from Ensembl Bacteria; the complete DNA sequence (*_dna.toplevel.fa) and the general feature format (GFF) file (*.gff3) containing the position of each gene. The current collection of CDS genes presented in the MO annotations from Ensembl [Current Ensembl Annotation (CEA)] was taken as the reference annotations for this study. Prokaryotic genomes exhibit high levels of gene density, often with little extraneous DNA, which is ‘commonly perceived as evidence of adaptive genome streamlining’ (Sela *et al.*, 2016). Unannotated DNA represents between ∼10% and 20% of the six MO genomes selected and while an additional 0.38–2.22% is attributed to non-coding annotations, there is still a measurable portion of each genome without any annotation. This study focuses specifically on the identification of CDSs, which constitute the significant majority of annotated genomic regions in the six genomes studied (82.76–90.62%, see Table  1).

The CDSs from each of the six genomes exhibit a range of differences, which are known to impact the ability of prediction tools to identify them. These include, but are not limited to, GC content, codon usage and gene length. The GC content varies from 31.69% to 67.21% for these genomes, and even within a single genome, the CDS GC content varies widely (see Supplementary Fig. S1 for distributions). Furthermore, the canonical ATG start codon is used between 68.58% and 90.67% of the genes for the six genomes (see Supplementary Table S1 for more detail).

Additionally, *M.genitalium* uses the codon translation table 4, meaning one of the three universal stop codons (TGA/UGA) is instead used to code for tryptophan (Dybvig and Voelker, 1996), whereas the other five MOs use the universal translation table 11 (see Supplementary Tables S1 and S3 for more detail). While a similar median CDS length is shared across the six genomes, *B.subtilis* and *P.fluorescens* have a number of long genes (>8000 nt, see Supplementary Fig. S2) and *S.aureus* contains the 31,421nt ‘giant protein Ebh’ (Cheng *et al.*, 2014), which is more than twice the length of the next largest CDS in this study. The diversity across the rest of prokaryotes is likely to be as great as, or greater than, reported here for these six.

The Sequence Ontology (Eilbeck *et al.*, 2005) describes an ORF as ‘The in-frame interval between the stop codons of a reading frame which when read as sequential triplets, has the potential of encoding a sequential string of amino acids’. However, it is conventional for ORFs to be reported as regions of DNA encompassed by a start and stop codon as a start codon is expected to indicate the start of DNA transcription (Brent, 2005). We acknowledge the difference in ontological definition and during this study, we refer to the region of DNA between an in-frame start and stop codon that is predicted to encode for an amino acid (protein) sequence, as a predicted CDS.

### 2.2 Prediction tools

This study specifically investigates CDS predictors, tools which apply complex filtering after the identification of ORFs across a region of DNA. This is different to ORF finders, which return unfiltered ORFs (Stothard, 2000) that meet a set of pre-defined rules, such as length and in-frame start and stop codons. This filtering is unique to each tool and dependent on properties, such as codon usage, GC content, CDS length, overlap and similarity to known genes and other more sophisticated parameters modelled on analysis of previously studied genes and genomes. Without such filtering methods, CDS predictors would typically report many false positives, such as nested or heavily overlapping CDSs. An example of filtering can be found in in the GeneMark (Borodovsky and McIninch, 1993) algorithm, which reports multiple variations of the same CDS with confidence scores. For this study, we chose the longest for each CDS after assessing the results.

We selected 15 different CDS prediction tools, some of which required a model (a rigid set of parameters adjusted to a particular organism), and the others, which predicted *ab initio* from sequence. The tools, which required a model were: GeneMark.hmm with *E.coli* and *S.aureus* models (Lukashin and Borodovsky, 1998); FGENESB with *E.coli* and *S.aureus* models (Salamov and Solovyevand, 2011); Augustus with *E.coli*, *S.aureus* and *Homo* *sapiens* models (Keller *et al.*, 2011); EasyGene with *E.coli* and *S.aureus* models (Nielsen and Krogh, 2005); GeneMark with *E.coli* and *S.aureus* models (Borodovsky and McIninch, 1993). Those which did not require a model were: GeneMarkS (Besemer *et al.*, 2001); Prodigal (Hyatt *et al.*, 2010); MetaGeneAnnotator (Noguchi *et al.*, 2008); GeneMarkS-2 (Lomsadze *et al.*, 2018); MetaGeneMark (Zhu *et al.*, 2010); GeneMarkHA (Besemer and Borodovsky, 1999); FragGeneScan (Rho *et al.*, 2010); GLIMMER-3 (Delcher *et al.*, 2007); MetaGene (Noguchi *et al.*, 2006); and TransDecoder (Haas *et al.*, 2013). The two groups are referred to as ‘model-based’ and ‘*ab initio*’ henceforth and can be seen in Table  2. The group *ab initio* included a number of tools, which were designed for fragmentary and metagenomic studies: MetaGeneMark, MetaGene, MetaGeneAnnotator and FragGeneScan. In addition, TransDecoder was developed to predict coding regions within transcript sequences, often in eukaryotes. To emulate the annotation process of a novel or less studied genome or metagenome, each tool was run using its default parameters. More information regarding each group and tool, and the parameters used to run them, can be found in Supplementary Section S3 ‘Prediction Tools’.

**Table 2.** Version number and reference for all tools used in this study

No.	Tool name	Version	Reference
1	Augustus	3.3.3	Keller *et al.* (2011)
2	EasyGene	1.2	Nielsen and Krogh (2005)
3	GeneMark.hmm	3.2.5	Lukashin and Borodovsky (1998)
4	GeneMark	2.5	Borodovsky and McIninch (1993)
5	FGENESB	‘2020’	Salamov and Solovyevand (2011)
6	Prodigal	2.6.3	Hyatt *et al.* (2010)
7	GeneMarkS	4.25	Besemer *et al.* (2001)
8	GeneMarkS 2	‘2020’	Lomsadze *et al.* (2018)
9	GLIMMER 3	3.02	Delcher *et al.* (2007)
10	GeneMark (H.A)	3.25	Besemer and Borodovsky (1999)
11	TransDecoder	5.5.0	Haas *et al.* (2013)
12	FragGeneScan	1.3.0	Rho *et al.* (2010)
13	MetaGene	2.24.0	Noguchi *et al.* (2006)
14	MetaGeneMark	‘2020’	Zhu *et al.* (2010)
15	MetaGene Annotator	2008/8/19	Noguchi *et al.* (2008)

*Note*: Tools 1–5 inclusive are model-based tools. Tools 6–15 inclusive are *ab initio-*based tools. Where no version number is available, the year when the tool was used is listed in single quotes.

Whole genome annotation ‘pipelines’, such as PROKKA (Seemann, 2014) and NCBI’s PGAP (Tatusova *et al.*, 2016) were not included, but the initial CDS prediction components embedded in these pipelines, such as Prodigal and GeneMarkS-2, were included in the study. Multiple separate tools from the GeneMark family (Besemer and Borodovsky, 2005) were included (some superseded) due to their extensive use and impact on genomic knowledge over the last three decades.

### 2.3 Comparison method

A systematic software platform ORForise (ORF Authorise) was built to perform a fair, comparative, and informative analysis of the different tools examining different aspects of their predictions. Version 1.0 of the platform, written in Python3 (Van Rossum and Drake, 2009), was used and is freely available at https://github.com/NickJD/ORForise. It has been designed to process the standardized GFF3 format as well as the individual output formats produced by each tool listed in this study.

**Fig. 1.**
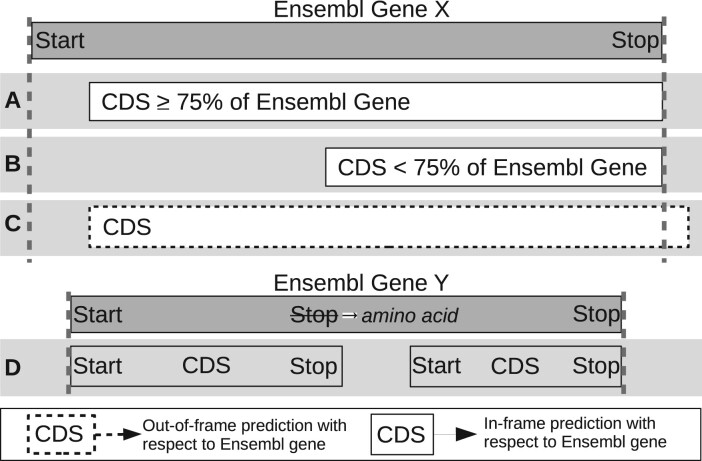
Illustration of how predicted CDSs are classified as having detected or not detected the CEA genes. Predicted CDSs are compared to the genes held in Ensembl. (**A**) The predicted CDS covers at least 75% and is in-frame with Ensembl gene and therefore it is recorded as detected. (**B**) The predicted CDS covers <75% of the Ensembl gene and therefore is recorded as not detected. (**C**) The predicted CDS covers part of an Ensembl gene but is out of frame (dotted outline) and therefore is recorded as missed. (**D**) The use of alternative stop codons causes the predicted CDS to be truncated or divided into two CDSs that span the Ensembl genes and therefore is recorded as missed

In this platform, we endeavoured to choose a wide range of metrics that clearly and representatively capture the many intricacies of the predictions. A number of metrics used in previous studies, such as the number of CDSs predicted, accurate identification of start positions or the number of genes correctly detected, can give some indication of the ‘accuracy’ of each tool. However, it was found during our analysis that there were many complexities in the prediction results, which would not be represented by these high-level metrics. For example, predicted CDS regions may overlap with one or more known CEA genes but be inaccurately extended or truncated on either the 5' or 3' end. It is also common for smaller CEA genes to be mistakenly encompassed by larger predicted CDSs and while the nucleotide regions of these genes are technically within the predicted regions, even if in-frame, they do not represent the true protein-CDS. Furthermore, different types of inaccuracies may be more or less important, depending on the aim of any given study. Therefore, clear and specific measures of accuracy that describe the detection of the entire locus of a gene are needed. Figure  1 illustrates how we determine correct CEA gene detection, but also explains its nuances and complexities. An example of this is the definition of short ORFs, which in prokaryotes are often described as having lengths of 100–300 nt (Duval and Cossart, 2017; Storz *et al.*, 2014; Su *et al.*, 2013). However, due to hard-coded cutoffs in many of the tools, we chose the ‘upper-bound’ of 300 nt or 100 codons to define short ORFs. We iteratively developed 72 metrics to help provide the most accurate and informative representation of a tool’s prediction quality. Additionally, as part of the ORForise platform, we provide a number of Python3 post-analysis scripts developed to aid in the interrogation between the CEA gene annotations and the CDSs predicted by each of the tools studied. These scripts were used to extract characteristics that are useful in the investigation of why specific CEA genes are detected, missed or incorrectly reported.

### 2.4 Aggregated tool predictions

An extension to the ORForise comparison platform was built (Aggregate_Compare) to investigate whether an aggregation of predictions from a number of top-performing tools would perform better than individual tools. The CDS predictions from the selected tools are combined into a single data structure with duplicate CDSs filtered out, but alternative predictions for the same locus retained and ordered according to start position. The same comparison algorithm could then be employed on the set of unique CDS predictions identified by this union of the outputs of the selected tools (Prodigal, GeneMark-S-2, MetaGeneAnnotator, MetaGeneMark and GeneMark-S—chosen due to their individual performance) and as with the singular tool comparison, for every CEA gene, the CDS, which deviated the least from the correct locus was selected as the closest match.

### 2.5 Discovering additional ORFs

To enable the aggregation of different CDSs from contemporary and new annotations, we provide GFF_Intersector to create a single GFF representing the intersection of two existing annotations. This also provides an option to allow the retention of CDSs that have a user-defined difference (default minimum 75% coverage and in-frame). Additionally, we also provide the GFF_Adder tool, which produces a new GFF containing CDSs from an existing annotation, plus the new CDSs, filtered to remove any that overlap existing CDSs by more than 50 nt (user definable).

## 3 Results

### 3.1 Metrics for comparison of tools

A total of 72 different metrics were chosen for this exhaustive evaluation in order to give the broadest possible scope to compare and contrast the performance of the tools. The full definitions for each of these metrics can be found in Supplementary Section S5 and are intended to be used as a resource for the community when deciding which tool to apply to both novel and contemporary genome annotation work. The following are 12 of the most informative metrics, selected for their ability to represent both a broad range and depth of different attributes which have been used to distinguish the prediction tools.

- **M1** Percentage of Genes Detected
- **M2** Percentage of Predicted CDSs that Detected a Gene
- **M3** Percentage Difference of Number of Predicted CDSs
- **M4** Percentage Difference of Median Predicted CDS Length
- **M5** Percentage of Perfect Matches
- **M6** Median Start Difference of Matched Predicted CDSs
- **M7** Median Stop Difference of Matched Predicted CDSs
- **M8** Percentage Difference of Matched Overlapping Predicted CDSs
- **M9** Percentage Difference of Matched Short Predicted CDSs
- **M10** Precision
- **M11** Recall
- **M12** False Discovery Rate

M1, Percentage of Genes Detected, is often used as the main indicator of tool performance in other comparisons but interpreted differently between studies. Here, it is characterized as a predicted CDS, which is in frame with a CEA gene and has captured at least 75% of its nucleotide sequence (Fig.  1A). In contrast to M1, which indicates when underprediction (or false negatives) occurs, M2 suggests when overprediction (or false positives) has occurred.

For M3, M4, M8 and M9, *Percentage Difference* was used to identify differences between predicted and CEA metrics: 100*(Predicted CDS metric—Ensembl Gene Metric)/Ensembl Gene Metric. The best score for a metric using the *Percentage Difference* calculation is 0, as 0 represents no deviation from the CEA annotations. The ‘Matched CDSs’ identifier used for M6, M7, M8 and M9 represent the CDSs, which have correctly detected an CEA gene. M6 and M7 are calculated by taking the median codon position differences recorded for mispredicted start or stop codons. Metrics, such as the Percentage of Perfect Matches (M5) can give a clearer overview of a tool’s ‘accuracy’ or performance, as it is common for a tool to misidentify either the exact start or stop locus of a detected CEA gene, while metrics, such as Median Start Difference of Matched Predicted CDSs (M6) can help establish the level of inaccuracy.

**Fig. 2.**
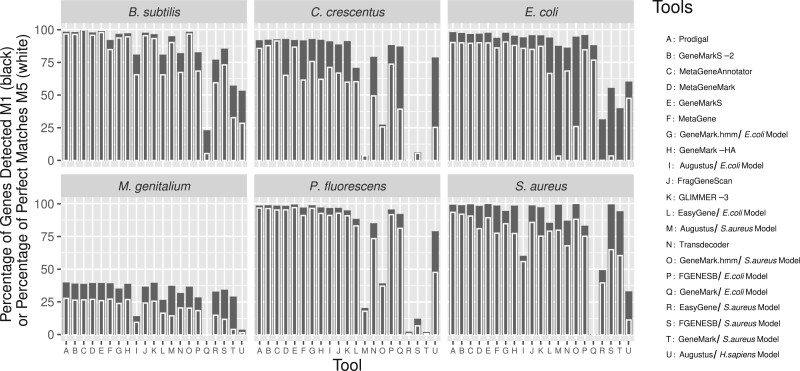
The result of all 15 gene prediction tools (21 with chosen models) on the 6 MO genomes, ordered by the summed ranks across the 12 metrics. The *Y* axis represents the Percentage of Genes Detected (M1) by each tool in black and the Percentage of Perfect Matches (M5) in white. M5, which represents the ability for a tool to detect the correct start codon, has more variance between the tools than M1. Each column on the *X* axis represents a different tool (some model-based tools were run multiple times). There is considerable variation in how well each tool performs across the different genomes, while all tools perform relatively poorly on the *M.genitalium* genome

The tools were ordered by totalling the rankings for each of these 12 metrics, across the 6 MOs. Supplementary Results S1 contains the results used for the ranking. This ranking, based on a wide range of different performance measures, allows for a comparative overview of contemporary and future tools, and is presented in Figure  2. This figure also shows the Percentage of Genes Detected (M1) with an overlay of the Percentage of Perfect Matches (M5), demonstrating the inconsistency between the two metrics for each tool. Metrics, such as Percentage of Genes Detected (M1) and Percentage of Predicted CDSs that Detected a Gene (M2), are informative and can be representative of a tool’s prediction quality, however, they do not convey the complete picture when presented in isolation. This is of particular importance for those working with metagenomic or other fragmentary assemblies, as the likelihood of incomplete fragments and chimeric sequences is higher and can lead to varying mispredictions. Although the overall prediction quality of genes was high across most of the tools and genomes in this study, the additional metrics produced can be used to identify strengths and weaknesses inherent to them. For example, GeneMark.hmm (*S.aureus* model and genome), MetaGeneMark and MetaGeneAnnotator, GeneMarkS were all ranked highest for Percentage of Genes Detected (M1) for at least one MO, while Prodigal and GeneMarkS were ranked highest twice (GeneMarkS and GeneMark.hmm were ranked joint highest for *S.aureus*). However, when inspecting the 12 metrics (Supplementary Fig. S3), it was clear that there were complex differences between the prediction results of not only the highest scoring tools, but also the lower ranked tools, which were often ranked high for some metrics in some of the genomes.

While no tool or group of tools consistently ranked highest or equally across the 12 metrics or MOs, MetaGeneAnnotator ranked best for *B.subtilis* and *M.genitalium*, GeneMarkS-2 ranked best for *C.crescentus* and Prodigal ranked best for *E.coli*, *P.fluorescens* and *S.aureus*.

The combination of multiple metrics can be used to determine which tool should be used between two candidate tools with the same or similar Percentage of Genes Detected (M1). For *M.genitalium*, both GeneMarkS and MetaGeneMark obtained an M1 score of 39.50%, but MetaGeneMark reported a higher Percentage of Perfect Matches (M5) (65.96% compared to 61.17%) than GeneMarkS (see Fig.  2) and is thus more accurate.

In addition, GeneMarkS is ranked first for Percentage of Genes Detected (M1) when applied to *P.fluorescens* with 99.29%, compared to Prodigal, which is ranked 4th with 98.49%. However, Prodigal has the highest Percentage of Perfect Matches (M5), 92.86% versus 87.03% for GeneMarkS, which means that more of the CEA genes identified by Prodigal were exact matches. In this instance, choosing either Prodigal or GeneMarkS as the overall highest performing tool is not arbitrary.

### 3.2 Model-based versus *ab initio* tools

It was evident that the performance of model-based tools was less consistent across the six MOs than the *ab initio* tools. They could perform as well as or better than a number of *ab initio* tools when the model selected was the same as the genome annotated. However, if genome and model were not the same, they often produced predictions of extremely low quality. For example, GeneMark with the *E.coli* model only predicted 71 CDSs for *S.aureus’*s 2478 CEA genes, of which only 18 CDSs detected a CEA gene. However, while it could be expected that mixing different models and genomes could cause poor quality predictions from model-based tools, there were instances in which both model and genome were the same and the prediction performance was also poor. In particular, in the case of EasyGene using the *S.aureus* model, only 49.31% of *S.aureus* CEA genes were detected, a contrast from the ∼99% detected by the majority of *ab initio* tools.

Intriguingly, Augustus (a model-based tool) when employed with the *E.coli* model, was able to detect 96.64% of *P.fluorescens* genes. Both genomes are *Gammaproteobacteria*, and thus Augustus may be identifying common features of their genes. While this shows that model-based tools can perform well even when their model and target genomes are different, when Augustus was applied to *S.aureus* using the *S.aureus* model, it was only able to detect 20.53%, but unexpectedly detected 78.91% when using an *H.sapiens* model. This is indicative of the inconsistency of model-based prediction tools and the genome models they employ. In contrast, through the ranking approach, we employed, the model-based tool GeneMark.hmm with the *E.coli* model ranked higher (7/21) than a number of *ab initio* tools in both the overall ranking and for individual metrics. Furthermore, GeneMark.hmm with the *S.aureus* model was joint top in detecting the highest number of *S.aureus* CEA genes with GeneMarkS. Additionally, for each of the model-based tools, the *E.coli* model performed better across the six MOs than the *S.aureus* model.

### 3.3 GC content

No significant variation was observed between the CEA gene median GC content and that of the predicted CDSs from each tool, even for those with poor predictions (see Supplementary Results S2). As can be seen in Supplementary Figure S1, each of the six genomes exhibits CEA genes with a wide range of GC content profiles, irrespective of their genome’s median value. We note that the GC content of genes missed by Prodigal is lower for all six MOs, but within the 25–75th percentile range for all CEA genes (Supplementary Fig. S1 and Table S4). Notably, *E.coli* and *P.fluorescens* genes, which were missed by Prodigal are nearly 10% lower in GC content than both detected and partial genes.

### 3.4 Overlapping CDSs

The overall number of CDSs predicted to have an overlap with another CDS varied across each of the tools and MOs, with cases of both positive and negative percentage differences when compared to the CEA annotations (see Supplementary Results S2 ‘Full Prediction Metrics’). Proportionally, the number of overlapping CDSs reported by *ab initio* tools are closer to the number of overlapping CEA genes than those reported by the model-based group.

Most model-based tools underpredict the proportion of overlapping CDSs with the exception of GeneMark *E.coli* for *P.fluorescens*, which predicted 2073 overlapping CDSs compared to the 1251 reported by Ensembl (see Supplementary Tables S5 and S6 and result  S1  and  S2).

Correct detection of CEA overlapping genes is also a problem. By totalling and averaging the Percentage Difference of Matched Overlapping Predicted CDSs (M8), we were able to observe a clear difference between the two tool groups with respect to their ability to detect correct overlapping CEA genes (see Supplementary Tables S5 and S6). The inability of the tools to account for the unusual nature of the *M.genitalium* genome was shown again with an average M8 across all tools of −88.21%, compared to the average of −27.77% for the other five genomes.

Furthermore, when making predictions for *E.coli*, while model-based tools, such as Augustus and EasyGene with the *E.coli* model can closely predict the proportion of overall overlapping CDSs (Percentage Difference of −1.42% and −2.30%, respectively), due to the poorer performance of these tools for correctly detecting CEA genes, their M8 scores for matched overlapping CDSs were substantially lower than the average score of the *ab initio* tools (grouped average of −52.89% as opposed to −23.62%—see Supplementary Table S6). Prodigal exemplifies this difference between the two tool groups. It was able to predict all overlapping CEA from *P.fluorescens* and *S.aureus*, whereas even when paired with the same model and genome, model-based tools continued to perform poorly.

### 3.5 Short ORFs

The lengths of detected, partially matched and missed CEA genes when predicted by Prodigal are summarized in Supplementary Figure S4. It shows that the CEA genes, which were missed by Prodigal for each genome were substantially shorter in length than the genes, which were detected, except for *M.genitalium*. For the other five MOs, whose combined median length of missed genes is 317, less than half the combined median length of 837.5 of those detected (Supplementary Fig. S4 and Results S2), it is alternative start codon selection, which influences whether a predicted CEA is shortened or elongated.

The proportion of short CEA genes in the six genomes below 300 nt ranged from 4.8% to 13.6% for each of the six MOs. All tools predicted many short CDSs for *M.genitalium* because they were incorrectly truncated due to its alternative stop codon usage. On average, *ab initio* tools were shown to be more likely to correctly detect short CEA genes across the other five MOs (see Supplementary Tables S7 and S8). Interestingly, unlike overlapping genes, short ORFs were more often overpredicted, but few were actually accurate when compared to the CEA. However, *ab initio* tools were much better suited to reporting the correct proportion of short predicted CDSs for all six genomes, often reporting the same proportion (see Supplementary Table S7). While *M.genitalium* does exhibit the highest divergence in proportional reporting of short predicted CDSs, *ab initio* tools were still less divergent (see Supplementary Table S9).

### 3.6 Partial matches

The number of missed CEA genes was low across the tools studied, with the exception of *M.genitalium* and some outliers from the model-based tools, such as GeneMark, Augustus and EasyGene. However, we also found many genes that were incorrectly reported on the 5' or 3' end. These misannotations, which we have called ‘partial matches’ if in the correct frame and accounting for ≥75% of a CEA gene, constitute either an elongation or truncation of the protein product of the gene and therefore potentially have an unknown impact on the resultant sequence. A large number of genes were incorrectly reported on the 3' end for *M.genitalium* by each tool. These 3' truncated CDSs are explained by the alternative use of TGA as tryptophan in *M.genitalium* (tools incorrectly assume this encodes a stop codon). The stop codons predicted for *M.genitalium* by all the prediction tools were the same ‘TGA, TAG, TAA’ as for the CEA genes of the other five MOs. Interestingly, one CEA gene in *E.coli*, which used CTT as a stop codon, was missed by all tools except for FGENESB with its *E.coli* model. FGENESB incorrectly reported the very next codon, a TGA, as the stop position. This 78 nt CEA is the only example, we found of a tool extending a CEA gene not from *M.genitalium*. Augustus with the Human model made a number of non-standard predictions due to its propensity to search for multiple CDSs for each gene but this is to be expected and is not reported in these results. Unlike 3' misprediction, a large number of genes from all six genomes were predicted with alternative start codons (see Supplementary Results S2). This was true for all tools and especially a problem for *C.crescentus* with a relatively low 68.58% ATG start codon usage for all CEA genes. The CEA genes for which Prodigal was unable to obtain a ‘Perfect Match’ (M5), was just 37.40%. Prodigal used a much higher level of ATG (80.87%) for this set of partially matches genes. This misidentification of start codon usage was a consistent problem among all the tools and genomes studied. However, for *E.coli*, the level of misidentification was lower. As an example, the number of times the correct or incorrect start codons were selected by Prodigal, across all six MOs, including the number of incorrectly chosen instances of the start codon (e.g. a different ATG further upstream of the real ATG) can be seen in Supplementary Table S2.

### 3.7 Aggregated tool predictions

Combined prediction approaches have previously been utilized to harness the prediction power from multiple tools to increase the number of detected CEA genes (Tatusova *et al.*, 2016; Yok and Rosen, 2011). For each of the MOs, taking the union of the top 5 tool predictions did provide a small increase in the number of Genes Detected (M1) (and a reduction of partial matches) compared to that of the ‘best tool’ [tool with highest percentage of Genes Detected (M1)] for any particular organism. However, even with this extreme case of using the union of all predicted CDSs, the increase in M1 was negligible (average increase of 0.47%) and came at the expense of predicting a large number of additional incorrect CDSs, as can be seen in Supplementary Table S10. Even in the case of *M.genitalium*, the M1 was not improved more than 0.21% with the union prediction.

### 3.8 Improving historic annotations

Using the GFF_Adder tool, we investigated the potential of Prodigal to add additional CDSs to the CEA annotations. There are more than 60 additional predicted CDSs that can be found for each of our MOs, and more than 270 for *E.coli* and *P.fluorescens* (see Supplementary Table S11).

## 4 Discussion

### 4.1 *Ab initio* tools usually perform better than model-based

We found that *ab initio* tools usually perform better than model-based tools. While no one tool performed the best or worst across all metrics, the *ab initio* tools Prodigal, GeneMarkS-2, MetaGeneAnnotator, MetaGeneMark and GeneMarkS were ranked first–fifth, respectively, across our 12 metric ranking (Supplementary Fig. S3 and Results S1 and Supplementary Rankings).

Strains of the same species can exhibit large intraspecies variation (Van Rossum *et al.*, 2020). Additionally, genes resulting from horizontal transfer, which is more frequent within species (Van Rossum *et al.*, 2020), are likely to contain features from the donor strains, which the rigid model-based methods are unable to recognize. GeneMark, a model-based tool, published in 1993, even when both target genome and model were *E.coli*, was identified as one of the worst performing tools in this study, possibly driven by the well-known large open pangenome of this species (Lukjancenko *et al.*, 2010). The same was observed for *S.aureus*. While model-based tools can perform well even when their model and target genomes are different, in the case of Augustus, when applied to the *C.crescentus* genome using the *S.aureus* model, it was only able to detect 3.93% of CEA genes, but unexpectedly detected 78.75% when using the *H.sapiens* model. Unsurprisingly, model-based predictors have therefore fallen out of development and use over the last decade and *ab* *initio*-based tools, such as Prodigal, GeneMarkS-2 and GLIMMER3 have become ubiquitous.

### 4.2 Codon usage has a large influence on accuracy

We found that codon usage has a large influence on accuracy due to its influence on start and stop codon choice, even in MOs.

The re-coding of a stop codon as an amino acid is rare and seems to be taxa specific (Dybvig and Voelker, 1996). While many of the tools offered the ability to change codon tables (often accounting for TGA specifically), the correct codon tables or codon preferences for each genome cannot be known in advance of annotation of a novel organism. Despite this, we would expect that they should be able to predict a significant proportion of genes, even in the absence of the knowledge of a different codon usage table. Some tools, such as Prodigal will assess a genome using both the universal and *Mycoplasma* translation table, however remarkably this did not increase the accuracy of the tool when analysing *M.genitalium* genome (see Fig.  2). Overall TGA was never predicted as tryptophan-coding in this genome by any tool (see Supplementary Results S2).

While ATG is used for 80% of start codons in the canonical annotations for most prokaryote genomes, some species and even some species-spanning gene families have been shown to use very different start codon profiles (Villegas and Kropinski, 2008). The use of different start codons in prokaryote genomes has often been correlated to the genome-wide GC content: at extreme low and high GC (<30% and >80%), ATG and GTG, respectively, are often more prominent. In our study, the extreme example of this was *C.crescentus*, which uses ATG as a start codon only 69% of the time. This is likely driven by its GC content of 67%. All of the tools performed poorly at predicting the correct start codon in this species (Fig.  2). This has been reported in the literature, specifically in relation to the lack of translation initiation sequence motifs traditionally used by prediction tools to identify the frame and start locus of a gene (Schrader *et al.*, 2014). This is not unique to *C.crescentus* and as shown in Supplementary Table S2, for all six MOs incorrect start codon selection resulted in either elongated or truncated CDSs (see Supplementary Results S2). The analysis of *E.coli* exhibited the lowest divergence between CEA and predicted start codon selection (see Supplementary Results S2 for more detail), possibly as a result of its historic use as a MO and having the largest use of the canonical ATG start codon in this study. Studies continue to investigate the possible fluidity of gene start codon selection and how some genes recorded in genomic databases may either have been annotated with the wrong start codon, or even require the annotation of multiple alternative start positions and therefore start codons (Baranov *et al.*, 2015; Meydan *et al.*, 2019; Villegas and Kropinski, 2008).

### 4.3 Metagenomic annotation approaches are suitable for whole genome sequences

Interestingly, tools made specifically for metagenomic and fragmented genome annotation performed better than most single genome tools (tools ranked third, fourth and sixth were developed for metagenome annotation), possibly indicating that even ‘complete’ genomes may themselves still harbour elements of sequencing and assembly error which these types of algorithms have been designed to account for. Most genomes submitted to databases, such as the NCBI Genome repository (Haft *et al.*, 2018), are incomplete and can contain hundreds of fragments which can make gene prediction an even more difficult task. As S. Salzberg said in 2019 ‘Paradoxically, the incredibly rapid improvements in genome sequencing technology have made genome annotation less, not more, accurate’ (Salzberg, 2019). This indicates that future annotation work performed on non-model and more diverse organisms may benefit from approaches implemented by metagenomic tools.

### 4.4 Short genes and overlapping genes are often misreported

We found that short genes and overlapping genes are often misreported and that many tools still have hard-coded limitations and weightings against these types of genes, with model-based tools performing especially poorly.

It has been well established in the literature that short genes are likely under-represented across genomic databases, and therefore, possibly even within the Ensembl data used in this study (Duval and Cossart, 2017; Storz *et al.*, 2014; Su *et al.*, 2013). The growing acceptance that short genes are not only common in prokaryotic genomes but also have important roles (Andrews and Rothnagel, 2014), is at odds with many tools still containing hard-coded limitations for minimum CDS length and algorithmic weights against short CDSs. As might be expected because of its re-coding of TAG, *M.genitalium* proved challenging for all tools to accurately identify CDSs, resulting in the early truncation of a large proportion of CEA genes and an increase in predicted short CDSs. This often led to the tools predicting additional spurious short CDSs in the missed regions (a result that can be seen in the low M10 Precision metric for this genome). However, for the other genomes, most tools also predicted too many short CDSs (9.07% and 39.10%, for *ab initio*- and model-based tools, respectively), but paradoxically still managed to miss a large proportion of Short CEA genes in the Ensembl annotations (missing 26.38% and 53.69% for *ab initio*- and model-based tools, respectively) (see Supplementary Tables S7–S9 and Results S2).

For overlapping genes, while *ab initio* tools performed better than model-based tools (see Supplementary Tables S5 and S6), in general they both under-predicted the number of overlapping CEA genes across the genomes (on average −6.07% and −30.15% for *ab initio*- and model-based tools, respectively) (see Supplementary Tables S5 and S6 and Results S2). No tool was able to correctly detect more than 20% of *M.genitalium’s* overlapping CEA genes. Overlapping and nested genes have now become an area of renewed interest for their potential impact on genomic organization and evolution (Huvet and Stumpf, 2014; Krakauer, 2000). For example, mokC in *E.coli*, believed to be a regulatory peptide, completely overlaps hokC and enables hokC expression (Pedersen and Gerdes, 1999) and no tool was able to detect both genes correctly.

Overall, the tools struggled to handle overlapping gene loci, and often returned either only one or neither of the overlapping coding regions in their predictions. This may be due to the manner in which many tools filter multiple candidate ORFs for a single locus leading to sub-optimal predictions. For example, Prodigal reports a CDS in *C.crescentus* on the positive strand at 23 760–24 074 when the CEA CDS is 23 550–24 170 on the negative strand. The unallocated space (24 074–24 170) resulted in Prodigal reporting the next downstream CDS starting at 24 091 instead of 24 133 (as in the Ensembl annotation), erroneously including 5' UTR in the predicted CDS. There are now tools to identify putative short ORFs in both prokaryotes and eukaryotes using additional evidence, such as RNA expression data (Bartholomäus *et al.*, 2021; Ji *et al.*, 2020; Miravet-Verde *et al.*, 2019). Our results suggest that the identification of short and overlapping CDSs cannot be done independently without the potential for unforeseen consequences for annotation accuracy.

### 4.5 Historic bias affects gene prediction today

Overall, we have observed an increase in accuracy in tools over time as can be seen with the different versions of GeneMark compared here: the overall rankings of model-based GeneMark (1993) (with *E.coli/S.aureus* models), *ab initio* GeneMarkS (2001) and *ab initio* GeneMarkS-2 (2018) are 20/17, 5 and 2, respectively. However, GeneMarkS (2001) performed better than its successor GeneMarkS-2 (2018) for 5 out of the 12 metrics in Supplementary Figure S3 including Percentage of Genes Detected (M1) in *P.fluorescens*, *M.genitalium* and *B.subtilis* (see Supplementary Results S1 and Supplementary Rankings). The performance of GeneMarkS (2001) in M1 may reflect its use for an extended period of time in the NCBI Prokaryote Annotation Pipeline. Possibly as a result of this, many of the CEA genes GeneMarkS (2001) detected were originally identified by the tool itself. Similarly, all model-based tools performed at their best across the 12 metrics and 6 MOs when using their *E.coli* model, hinting at the impact of historical research in this organism. Advances in the realms of machine learning and statistical modelling have the greatest potential to address these issues but are also likely to be the most prone to historical biases in the databases. Many of the rules, such as standard CDS length and codon usage, are inferred from previously identified CDSs. The existence of annotation errors and omissions in various sequence databases is well established and unlikely to be resolved in the near future without significant coordination between repositories (Klimke *et al.*, 2011). Additionally, much of the sequence information derived from MOs will become less relevant as greater numbers of novel organisms are sequenced (Hunter, 2008).

These issues have been raised previously: In 2009, the ‘Best Practices in Genome Annotation’ meeting report listed a number of areas of concern put forward by attendees (Madupu *et al.*, 2010) including tool choice, strategy to update and correct previous annotations, tracking of changes in databases, prioritization of certain genes for experimental evaluation, documenting processes and keeping up with technological advances. The work presented here addresses the issue of tool choice, but many of the recommendations are yet to be realized. The lack of any previous detailed systematic overview of method performance may also have played a part in these biases not being addressed to date. Our study has shown that tool selection needs to be fully informed by its intended purpose and the tool’s weaknesses.

### 4.6 Current and future techniques are needed to continue annotation improvements

It is clear from both this and previous studies that combinatory approaches are fundamental in bridging the gap to the next stage of genome annotation. This has clearly already begun with pipelines, such as PROKKA and PGAP, which utilize a collection of techniques, most notably, advanced homology searching to complete annotations where traditional CDS predictions fail or produce competing predictions. However, this can also lead to conflicting annotations. As noted, homology searches are only as good as the database being used. The presence or absence of homology does not indicate whether an ORF is a true CDS gene, especially in the nuanced field of alternative ORFs (Orr *et al.*, 2020). Further complications involving alternative ORFs, many of which are overlapping, can be found with new evidence in *E.coli*, where ‘Ribosome profiling revealed out-of-frame internal minimal ORFs in 13 *E. coli* genes. Mutation of the start codon… in one gene, yecJ, resulted in an increase in translation of the main-ORF, suggesting that these minimal ORFs also can modulate translation of the main-ORF’ (Meydan *et al.*, 2019).

As users of computational genomic techniques, we must realize when we have reached the limit of what is possible with the contemporary data available. This, together with other studies, proposes that the linchpin required for the next step in genome annotation, is not even more techniques reliant on current genomic knowledge, but instead more experimental work and species agnostic approaches. However, the near unlimited scope of growth conditions, environmental pressures et cetera, has made the prospect of experimentally validating all potential CDS regions unfeasible. Finally, while great strides have been made in experimentally validating difficult to characterize gene types, one such study ‘… suggest[s] substantial numbers of small proteins remain undiscovered in *E. coli*, and existing bioinformatics techniques must continue to improve to facilitate identification’ (VanOrsdel *et al.*, 2018).

## 5 Conclusion

We have presented a comprehensive set of metrics, which distinguish CDS prediction tools from each other and make it possible to identify which performs better for specific use-cases. The ORForise evaluation framework enables users to evaluate new and existing annotations and generate consensus and aggregate gene predictions. We have demonstrated that certain types of genes, such as short genes, overlapping genes and those with alternative codon usage, are still elusive, even to the most advanced *ab initio* techniques. Worryingly, the performance of any tool seems to depend on the genome that is being analysed. For instance, Prodigal, which ranked best overall, was ranked first for *E.coli*, *S.aureus* and *P.fluorescens*, MetaGeneAnnotater was ranked first for *B.subtilis* and *M.genitalium* and GeneMarkS-2 was ranked first for *C.crescentus* (see Supplementary Rankings). Additionally, no individual tool ranked as the most accurate across all genomes for the Percentage of Genes Detected (M1) (the single metric historically used to assess tool performance) or any other individual metric. This is likely to have a measurable impact on downstream genomic and pangenomic studies. However, overall, we found Prodigal to be one of the most well-rounded tools, not only detecting the highest number of CEA genes for two very diverse MOs (*E.coli* and *M.genitalium*), but also performing overall best when ranked across the 12 metric rankings and 6 MOs. It was also overall best for Perfect Matched genes (M5). However, as outlined earlier, it was not always ranked first for all genomes, further suggesting that users should choose tools carefully, based on the organism and question they are studying. Finally, we advise against generating aggregated *ab initio* annotations from multiple tools where no existing annotation is available for the genome, as this results in poor overall performance. However, additional cycles of annotation with tools designed to identify putative CDSs in the unannotated regions, show promise for improving current prokaryotic genomic knowledge.

## Author contributions

All authors discussed the conceptualization of the comparison platform and its impact. N.J.D. wrote the code. All authors contributed to the manuscript.

## Funding

This work was supported by an Institute of Biological, Environmental and Rural Sciences Aberystwyth PhD fellowship (to N.J.D.). C.J.C. wishes to acknowledge funding from the Biotechnology and Biological Sciences Research Council (BB/E/W/10964A01, BBS/OS/GC/000011B); Department of Agriculture, Food and the Marine Ireland/DAERA Northern Ireland (Meth-Abate, R3192GFS); and the European Commission via Horizon 2020 (818368, MASTER). 

*Conflict of Interest*: none declared. 

## Supplementary Material

btab827_Supplementary_Data

## References

[btab827-B1] Al-Turaiki, Israa M., et al. "Computational approaches for gene prediction: a comparative survey." *International Conference on Informatics Engineering and Information Science*. Springer, Berlin, Heidelberg, 2011.

[btab827-B2] Andrews S.J. , Rothnagel J.A. (2014) Emerging evidence for functional peptides encoded by short open reading frames. Nat. Rev. Genet., 15, 193–204. 24514441 10.1038/nrg3520

[btab827-B3] Badger J.H. , Olsen G.J. (1999) CRITICA: coding region identification tool invoking comparative analysis. Mol. Biol. Evol., 16, 512–524. 10331277 10.1093/oxfordjournals.molbev.a026133

[btab827-B4] Baranov P.V. et al (2015) Augmented genetic decoding: global, local and temporal alterations of decoding processes and codon meaning. Nat. Rev. Genet., 16, 517–529. 26260261 10.1038/nrg3963

[btab827-B5] Bartholomäus A. et al (2021) smORFer: a modular algorithm to detect small ORFs in prokaryotes. Nucleic Acids Res., 49, e89. 34125903 10.1093/nar/gkab477 PMC8421149

[btab827-B6] Besemer J. , Borodovsky M. (1999) Heuristic approach to deriving models for gene finding. Nucleic Acids Res., 27, 3911–3920. 10481031 10.1093/nar/27.19.3911 PMC148655

[btab827-B7] Besemer J. , Borodovsky M. (2005) GeneMark: web software for gene finding in prokaryotes, eukaryotes and viruses. Nucleic Acids Res., 33, W451–W454. 15980510 10.1093/nar/gki487 PMC1160247

[btab827-B8] Besemer J. et al (2001) GeneMarkS: a self-training method for prediction of gene starts in microbial genomes. Implications for finding sequence motifs in regulatory regions. Nucleic Acids Res., 29, 2607–2618. 11410670 10.1093/nar/29.12.2607 PMC55746

[btab827-B9] Borodovsky M. , McIninch J. (1993) GENMARK: parallel gene recognition for both DNA strands. Comput. Chem., 17, 123–133.

[btab827-B10] Brenner S.E. (1999) Errors in genome annotation. Trends Genet., 15, 132–133. 10203816 10.1016/s0168-9525(99)01706-0

[btab827-B11] Brent M.R. (2005) Genome annotation past, present, and future: how to define an ORF at each locus. Genome Res., 15, 1777–1786. 16339376 10.1101/gr.3866105

[btab827-B12] Browning D.F. , Busby S.J. (2004) The regulation of bacterial transcription initiation. Nat. Rev. Microbiol., 2, 57–65. 15035009 10.1038/nrmicro787

[btab827-B13] Burge C.B. , Karlin S. (1998) Finding the genes in genomic DNA. Curr. Opin. Struct. Biol., 8, 346–354. 9666331 10.1016/s0959-440x(98)80069-9

[btab827-B14] Cheng A.G. et al (2014) The giant protein Ebh is a determinant of *Staphylococcus aureus* cell size and complement resistance. J. Bacteriol., 196, 971–981. 24363342 10.1128/JB.01366-13 PMC3957702

[btab827-B15] Dalgarno L. , Shine J. (1973) Conserved terminal sequence in 18S rRNA may represent terminator anticodons. Nat. New Biol., 245, 261–262. 4202225 10.1038/newbio245261a0

[btab827-B16] Dandekar T. et al (1998) Conservation of gene order: a fingerprint of proteins that physically interact. Trends Biochem. Sci., 23, 324–328. 9787636 10.1016/s0968-0004(98)01274-2

[btab827-B17] Delcher A.L. et al (2007) Identifying bacterial genes and endosymbiont DNA with Glimmer. Bioinformatics, 23, 673–679. 17237039 10.1093/bioinformatics/btm009 PMC2387122

[btab827-B18] Devos D. , Valencia A. (2001) Intrinsic errors in genome annotation. Trends Genet., 17, 429–431. 11485799 10.1016/s0168-9525(01)02348-4

[btab827-B19] Dunne M.P. , Kelly S. (2017) OrthoFiller: utilising data from multiple species to improve the completeness of genome annotations. BMC Genomics, 18, 390. 28521726 10.1186/s12864-017-3771-x PMC5437544

[btab827-B20] Duval M. , Cossart P. (2017) Small bacterial and phagic proteins: an updated view on a rapidly moving field. Curr. Opin. Microbiol., 39, 81–88. 29111488 10.1016/j.mib.2017.09.010

[btab827-B21] Dybvig K. , Voelker L.L. (1996) Molecular biology of *Mycoplasmas*. Annu. Rev. Microbiol., 50, 25–57. 8905075 10.1146/annurev.micro.50.1.25

[btab827-B22] Eilbeck K. et al (2005) The sequence ontology: a tool for the unification of genome annotations. Genome Biol., 6, R44. 15892872 10.1186/gb-2005-6-5-r44 PMC1175956

[btab827-B23] Furnham N. et al (2012) Current challenges in genome annotation through structural biology and bioinformatics. Curr. Opin. Struct. Biol., 22, 594–601. 22884875 10.1016/j.sbi.2012.07.005

[btab827-B24] Guigo R. (1997) Computational gene identification: an open problem. Comput. Chem., 21, 215–222. 9415986 10.1016/s0097-8485(97)00008-9

[btab827-B25] Haas B.J. et al (2013) *De novo* transcript sequence reconstruction from RNA-seq using the Trinity platform for reference generation and analysis. Nat. Protoc., 8, 1494–1512. 23845962 10.1038/nprot.2013.084 PMC3875132

[btab827-B26] Haft D.H. et al (2018) RefSeq: an update on prokaryotic genome annotation and curation. Nucleic Acids Res., 46, D851–D860. 29112715 10.1093/nar/gkx1068 PMC5753331

[btab827-B27] Howe K.L. et al (2020) Ensembl Genomes 2020 – enabling non-vertebrate genomic research. Nucleic Acids Res., 48, D689–D695. 31598706 10.1093/nar/gkz890 PMC6943047

[btab827-B28] Hunter P. (2008) The paradox of model organisms: the use of model organisms in research will continue despite their shortcomings. EMBO Rep., 9, 717–720. 18670440 10.1038/embor.2008.142 PMC2515201

[btab827-B29] Huvet M. , Stumpf M.P. (2014) Overlapping genes: a window on gene evolvability. BMC Genomics, 15, 721. 25159814 10.1186/1471-2164-15-721 PMC4161906

[btab827-B30] Hyatt D. et al (2010) Prodigal: prokaryotic gene recognition and translation initiation site identification. BMC Bioinformatics, 11, 119. 20211023 10.1186/1471-2105-11-119 PMC2848648

[btab827-B31] Jain R. et al (1999) Horizontal gene transfer among genomes: the complexity hypothesis. PNAS, 96, 3801–3806. 10097118 10.1073/pnas.96.7.3801 PMC22375

[btab827-B32] Ji X. et al (2020) smORFunction: a tool for predicting functions of small open reading frames and microproteins. BMC Bioinformatics, 21, 1–13. 33054771 10.1186/s12859-020-03805-x PMC7559452

[btab827-B33] Kalkatawi M. et al (2015) BEACON: automated tool for Bacterial GEnome Annotation ComparisON. BMC Genomics, 16, 1–8. 26283419 10.1186/s12864-015-1826-4 PMC4539851

[btab827-B34] Keller O. et al (2011) A novel hybrid gene prediction method employing protein multiple sequence alignments. Bioinformatics, 27, 757–763. 21216780 10.1093/bioinformatics/btr010

[btab827-B35] Klimke W. et al (2011) Solving the problem: genome annotation standards before the data deluge. Stand. Genom. Sci., 5, 168–193. 10.4056/sigs.2084864 PMC3236044 22180819

[btab827-B36] Krakauer D.C. (2000) Stability and evolution of overlapping genes. Evolution, 54, 731–739. 10937248 10.1111/j.0014-3820.2000.tb00075.x

[btab827-B37] Land M. et al (2015) Insights from 20 years of bacterial genome sequencing. Funct. Integr. Genomics, 15, 141–161. 25722247 10.1007/s10142-015-0433-4 PMC4361730

[btab827-B38] Levy A. , Currie A. (2015) Model organisms are not (theoretical) models. Br. J. Philos. Sci., 66, 327–348.

[btab827-B39] Lobb B. et al (2020) An assessment of genome annotation coverage across the bacterial tree of life. Microb. Genom., 6, e000341. 32124724 10.1099/mgen.0.000341 PMC7200070

[btab827-B40] Lomsadze A. et al (2018) Modeling leaderless transcription and atypical genes results in more accurate gene prediction in prokaryotes. Genome Res., 28, 1079–1089. 29773659 10.1101/gr.230615.117 PMC6028130

[btab827-B41] Lukashin A.V. , Borodovsky M. (1998) GeneMark.hmm: new solutions for gene finding. Nucleic Acids Res., 26, 1107–1115. 9461475 10.1093/nar/26.4.1107 PMC147337

[btab827-B42] Lukjancenko O. et al (2010) Comparison of 61 sequenced *Escherichia coli* genomes. Microb. Ecol., 60, 708–720. 20623278 10.1007/s00248-010-9717-3 PMC2974192

[btab827-B43] Madupu R. et al (2010) Meeting report: a workshop on best practices in genome annotation. Database, 2010, baq001. 20428316 10.1093/database/baq001 PMC2860899

[btab827-B44] Mathé C. et al (2002) Current methods of gene prediction, their strengths and weaknesses. Nucleic Acids Res., 30, 4103–4117. 12364589 10.1093/nar/gkf543 PMC140543

[btab827-B45] Meydan S. et al (2019) Retapamulin-assisted ribosome profiling reveals the alternative bacterial proteome. Mol. Cell, 74, 481–493. 30904393 10.1016/j.molcel.2019.02.017 PMC7115971

[btab827-B46] Miravet-Verde S. et al (2019) Unraveling the hidden universe of small proteins in bacterial genomes. Mol. Syst. Biol., 15, e8290. 30796087 10.15252/msb.20188290 PMC6385055

[btab827-B47] Nielsen P. , Krogh A. (2005) Large-scale prokaryotic gene prediction and comparison to genome annotation. Bioinformatics, 21, 4322–4329. 16249266 10.1093/bioinformatics/bti701

[btab827-B48] Noguchi H. et al (2006) MetaGene: prokaryotic gene finding from environmental genome shotgun sequences. Nucleic Acids Res., 34, 5623–5630. 17028096 10.1093/nar/gkl723 PMC1636498

[btab827-B49] Noguchi H. et al (2008) MetaGeneAnnotator: detecting species-specific patterns of ribosomal binding site for precise gene prediction in anonymous prokaryotic and phage genomes. DNA Res., 15, 387–396. 18940874 10.1093/dnares/dsn027 PMC2608843

[btab827-B50] ÓhÉigeartaigh S.S. et al (2014) Searchdogs bacteria, software that provides automated identification of potentially missed genes in annotated bacterial genomes. J. Bacteriol., 196, 2030–2042. 24659774 10.1128/JB.01368-13 PMC4010983

[btab827-B51] Orr M.W. et al (2020) Alternative ORFs and small ORFs: shedding light on the dark proteome. Nucleic Acids Res., 48, 1029–1042. 31504789 10.1093/nar/gkz734 PMC7026640

[btab827-B52] Pedersen K. , Gerdes K. (1999) Multiple hok genes on the chromosome of *Escherichia coli*. Mol. Microbiol., 32, 1090–1102. 10361310 10.1046/j.1365-2958.1999.01431.x

[btab827-B53] Rho M. et al (2010) FragGeneScan: predicting genes in short and error-prone reads. Nucleic Acids Res., 38, e191. 20805240 10.1093/nar/gkq747 PMC2978382

[btab827-B54] Russell J.J. et al (2017) Non-model model organisms. BMC Biol., 15, 55–31. 28662661 10.1186/s12915-017-0391-5 PMC5492503

[btab827-B55] Salamov V.S.A. , Solovyevand A. (2011) Automatic annotation of microbial genomes and metagenomic sequences. In: Li, R.W. (ed.) Metagenomics and Its Applications in Agriculture. Nova Science Publishers, Hauppauge, pp 61–78.

[btab827-B56] Salzberg S.L. (2019) Next-generation genome annotation: we still struggle to get it right. Genome Biol., 20, 92. 31097009 10.1186/s13059-019-1715-2 PMC6521345

[btab827-B57] Schafer J.L. , Graham J.W. (2002) Missing data: our view of the state of the art. Psychol. Methods, 7, 147–177. 12090408

[btab827-B58] Schrader J.M. et al (2014) The coding and noncoding architecture of the *Caulobacter crescentus* genome. PLoS Genet., 10, e1004463. 25078267 10.1371/journal.pgen.1004463 PMC4117421

[btab827-B59] Seemann T. (2014) Prokka: rapid prokaryotic genome annotation. Bioinformatics, 30, 2068–2069. 24642063 10.1093/bioinformatics/btu153

[btab827-B60] Sela I. et al (2016) Theory of prokaryotic genome evolution. PNAS, 113, 11399–11407. 27702904 10.1073/pnas.1614083113 PMC5068321

[btab827-B61] Sommer M.J. , Salzberg S.L. (2021) Balrog: a universal protein model for prokaryotic gene prediction. PLoS Comput. Biol., 17, e1008727. 33635857 10.1371/journal.pcbi.1008727 PMC7946324

[btab827-B62] Stanke M. , Morgenstern B. (2005) AUGUSTUS: a web server for gene prediction in eukaryotes that allows user-defined constraints. Nucleic Acids Res., 33, W465–W467. 15980513 10.1093/nar/gki458 PMC1160219

[btab827-B63] Storz G. et al (2014) Small proteins can no longer be ignored. Annu. Rev. Biochem., 83, 753–777. 24606146 10.1146/annurev-biochem-070611-102400 PMC4166647

[btab827-B64] Stothard P. (2000) The sequence manipulation suite: JavaScript programs for analyzing and formatting protein and DNA sequences. Biotechniques, 28, 1102–1104. 10868275 10.2144/00286ir01

[btab827-B65] Su M. et al (2013) Small proteins: untapped area of potential biological importance. Front. Genet., 4, 286. 24379829 10.3389/fgene.2013.00286 PMC3864261

[btab827-B66] Tatusova T. et al (2016) NCBI prokaryotic genome annotation pipeline. Nucleic Acids Res., 44, 6614–6624. 27342282 10.1093/nar/gkw569 PMC5001611

[btab827-B67] Van Rossum G. , Drake F.L. (2009) Python 3 Reference Manual. CreateSpace, Scotts Valley, CA.

[btab827-B68] Van Rossum T. et al (2020) Diversity within species: interpreting strains in microbiomes. Nat. Rev. Microbiol., 18, 491–506. 32499497 10.1038/s41579-020-0368-1 PMC7610499

[btab827-B69] VanOrsdel C.E. et al (2018) Identifying new small proteins in *Escherichia coli*. Proteomics, 18, 1700064. 29645342 10.1002/pmic.201700064 PMC6001520

[btab827-B70] Villegas A. , Kropinski A.M. (2008) An analysis of initiation codon utilization in the Domain Bacteria–concerns about the quality of bacterial genome annotation. Microbiology, 154, 2559–2661. 18757789 10.1099/mic.0.2008/021360-0

[btab827-B71] Warren A.S. et al (2010) Missing genes in the annotation of prokaryotic genomes. BMC Bioinformatics, 11, 131. 20230630 10.1186/1471-2105-11-131 PMC3098052

[btab827-B72] Wood D.E. et al (2012) Thousands of missed genes found in bacterial genomes and their analysis with COMBREX. Biol. Direct, 7, 37–15. 23111013 10.1186/1745-6150-7-37 PMC3534567

[btab827-B73] Yok N.G. , Rosen G.L. (2011) Combining gene prediction methods to improve metagenomic gene annotation. BMC Bioinformatics, 12, 20. 21232129 10.1186/1471-2105-12-20 PMC3042383

[btab827-B74] Zhu W. et al (2010) *Ab initio* gene identification in metagenomic sequences. Nucleic Acids Res., 38, e132. 20403810 10.1093/nar/gkq275 PMC2896542

