# Supplementary material for: No one tool to rule them all: prokaryotic gene prediction tool annotations are highly dependent on the organism of study
Source: Bioinformatics. 2021 Dec 7;38(5):1198–207. doi: 10.1093/bioinformatics/btab827 (PMC8825762; doi:10.1093/bioinformatics/btab827)
Supplement: btab827_Supplementary_Data [file btab827_supplementary_data.zip › Supplementary.pdf]

Supplementary Information
No one tool to rule them all: Prokaryotic gene prediction tool annotations are highly dependent on the organism of study

Nicholas J. Dimonaco [1*], Wayne Aubrey [2], Kim Kenobi [3], Amanda Clare [2]†, and Christopher J. Creevey [4]†

[1]Institute of Biological, Environmental and Rural Sciences, Aberystwyth University, Aberystwyth, SY23 3PD, Wales, UK
[2]Department of Computer Science, Aberystwyth University, Aberystwyth, SY23 3PD, Wales, UK
[3]Department of Maths and Physics, Aberystwyth University, Aberystwyth, SY23 3PD, Wales, UK
[4]School of Biological Sciences, Queen's University Belfast, Belfast, BT7 1NN, Northern Ireland, UK

*To whom correspondence should be addressed. *nicholas@dimonaco.co.uk*
†The authors wish it to be known that, in their opinion, the last two authors should be regarded as Joint Senior Authors.

1 Model Organisms (Ensembl Bacteria Release 46)

- *Bacillus subtilis (B. subtilis) - Strain BEST7003 - Assembly ASM52304v1: B. subtilis* is a Gram-positive, genetically tractable, non-pathogenic model organism used in the industrial production of enzymes. It is part of the Firmicute phylum and is a useful model in the study of *Mycobacterium tuberculosis*, which is the the causative agent of tuberculosis. The strain BEST7003 with assembly ASM52304v1 was chosen for this study (Itaya *et al.*, 2005).

- *Caulobacter crescentus (C. crescentus) - Strain CB15 - Assembly ASM690v1: C. crescentus* is a Gram-negative, oligotrophic bacterium commonly found throughout freshwater lakes and streams. It is an important model organism for studying the regulation of the cell cycle, asymmetric cell division, and cellular differentiation and is part of the Proteobacteria phylum. The CB15 strain with the ASM690v1 assembly was chosen for this study (Nierman *et al.*, 2001).

- *Escherichia coli (E. coli) K-12 - Strain ER3413 - Assembly ASM80076v1: E. coli* is one of the most extensively studied microorganisms and is part of the Proteobacterium phylum. *E. coli* is Gram-negative and its genome was first completely sequenced in 1997. It was chosen then for its unique biochemical, molecular and biotechnological attributes but it widely studied now due to its tractability. The K-12 ER3413 strain with the ASM80076v1 assembly was chosen for this study (Anton *et al.*, 2015).

- *Mycoplasma genitalium (M. genitalium) - Strain G37 - Assembly ASM2732v1: M. genitalium* is a parasitic bacterium with one of the smallest currently known genomes of any free living bacterium at around 580,000 bps. Due to it being a human pathogen and its unique genome size, *M. genitalium* has been used as a model for a minimal organism in the study of essential genes due to being one of the most streamlined bacterial genomes currently known (Glass *et al.*, 2006). Although *M. genitalium* does not have cell walls, it is believed to have evolved from Gram-positive bacteria which had lost their cell wall and is part of the Firmicute phylum. The G-37 strain with ASM2732v1 assembly was chosen for this study (Hutchison *et al.*, 1999).

- *Pseudomonas fluorescens (P. fluorescens) - Strain UK4 - Assembly ASM73042v1: P. fluorescens* is a rod-shaped, Gram-negative bacterium and is part of the Proteobacteria phylum.The antibiotic Mupirocin can be produced by cultured *P. flurescens* and is used in the treatment of skin, ear and eye disorders and is a model organism for cell cycle, cell division and differentiation. The UK4 strain with the ASM73042v1 assembly was chosen for this study (Dueholm *et al.*, 2014).

- *Staphylococcus aureus (S. aureus) - Strain 502A - Assembly ASM59796v1: S. aureus* is Gram-positive bacterium of the Firmicute phylum and is commonly found on the human body, including the nose, skin and the respiratory tract. It has been known to cause diseases such as infective endocarditis and a drug resistant strain is commonly known as Methicillin-resistant *Staphylococcus aureus* (MRSA). The 502A strain with assembly ASM59796v1 was chosen for this study (Parker *et al.*, 2014).

2 Supplementary Figures

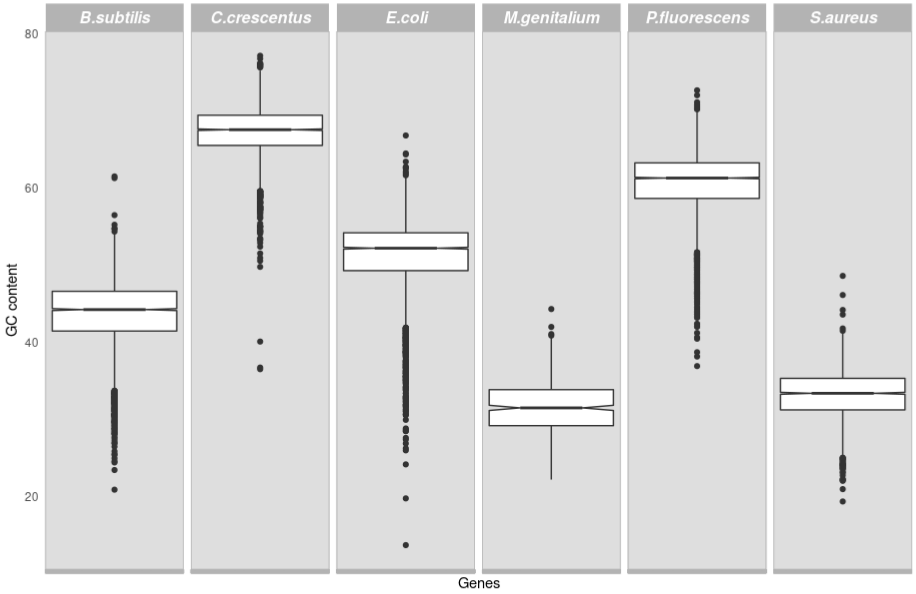

Figure 1: GC content of the six model organisms and their Ensembl annotated CoDing Sequences (CDSs). Note the high levels of variance within and between each genome.

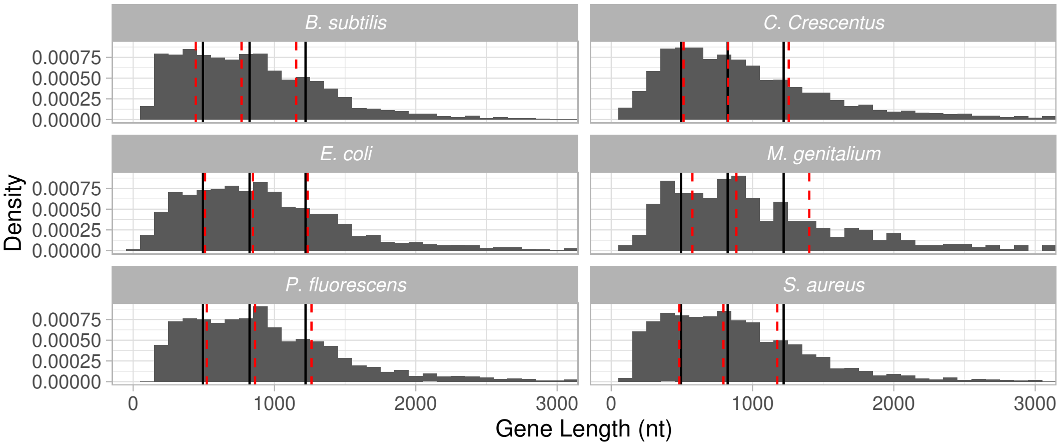

Figure 2: CoDing Sequence (CDS) lengths plotted for each model organism. The black, solid vertical lines are at the overall first quartile (494), median (824) and third quartile (1220) for all six model model organisms. The red dotted lines show the first quartile, the median and the third quartile for each organism individually. The x-axis is truncated at 3000 nt. The proportion of CDS lengths at or below this value are 0.964 for *M. genitalium*, 0.984 for *P. fluorescens*, 0.987 for *E. coli*, *S. aureus* and *C. crescentus*, and 0.990 for *B. subtilis*. A total of 23 CDSs were longer than 5000 nt. The distributions of CDS lengths for *E. coli*, *S. aureus*, *C. crescentus* and *P. fluorescence* are comparable to the overall distribution. The lengths for *B. subtilis* are somewhat smaller than expected overall, while the lengths for *M. genitalium* are longer than expected.

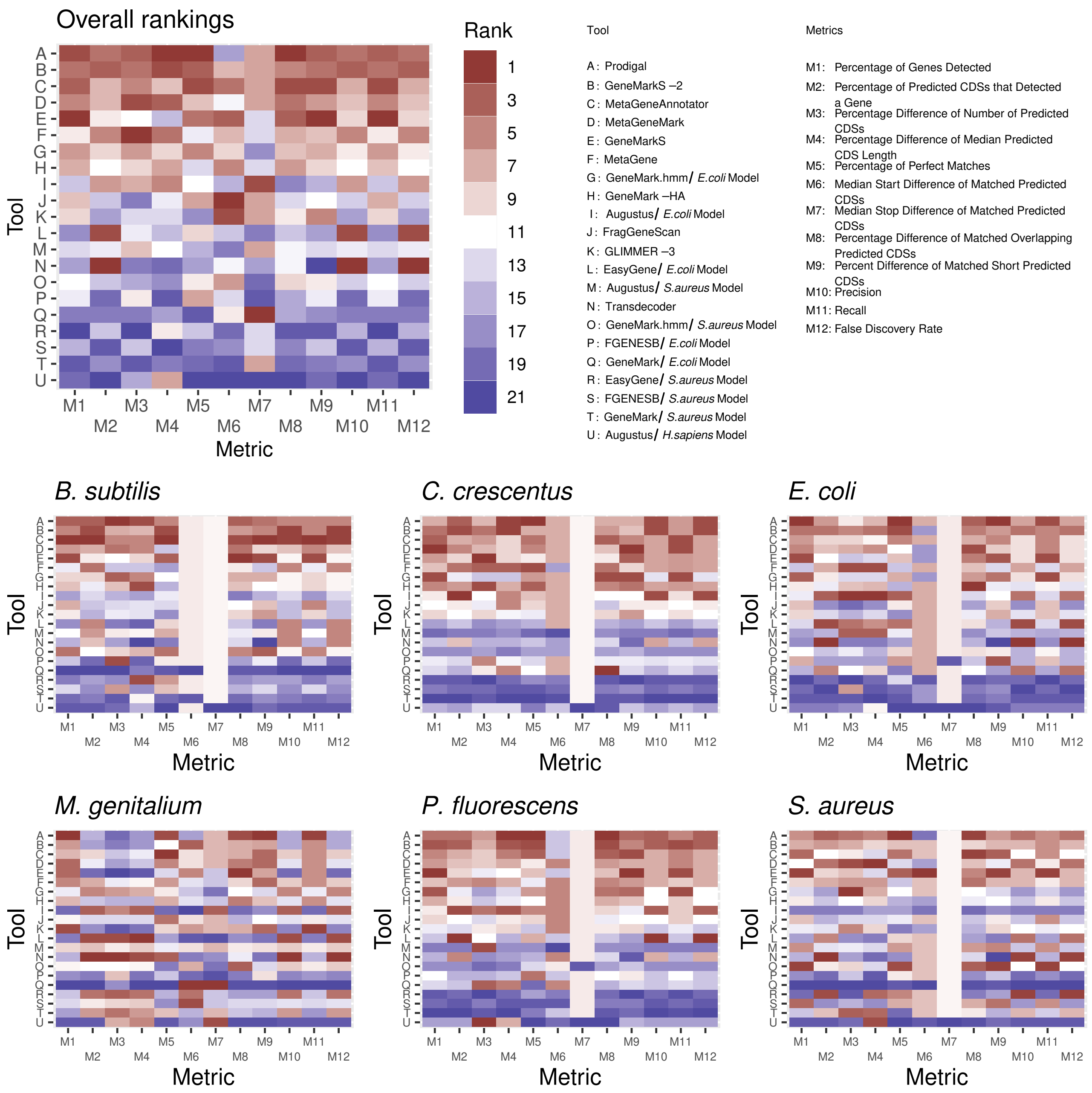

Figure 3: Heatmaps showing rankings of the tools by the 12 chosen metrics, overall and for each organism in turn. The tools are shown ordered by the summed ranks across the 12 metrics. While red is 'better' and blue is 'worse', it is clear that across the 6 model organisms, no tool stands out for these 12 metrics chosen as most representative. For example, for *C. crescentus*, GeneMark with *E. coli* model ranked 12th overall but reported the most accurate number of overlapping genes. For *P. fluorescens*, Prodigal was the overall highest ranked tool even though GeneMarkS detected the highest number of Ensembl genes. *M. genitalium* on the other hand, which uses an alternative stop codon, has some very interesting results showing the difficulty of identifying its genes by all tools. The pale coloured bands represent tools ranking the same for a particular metric.

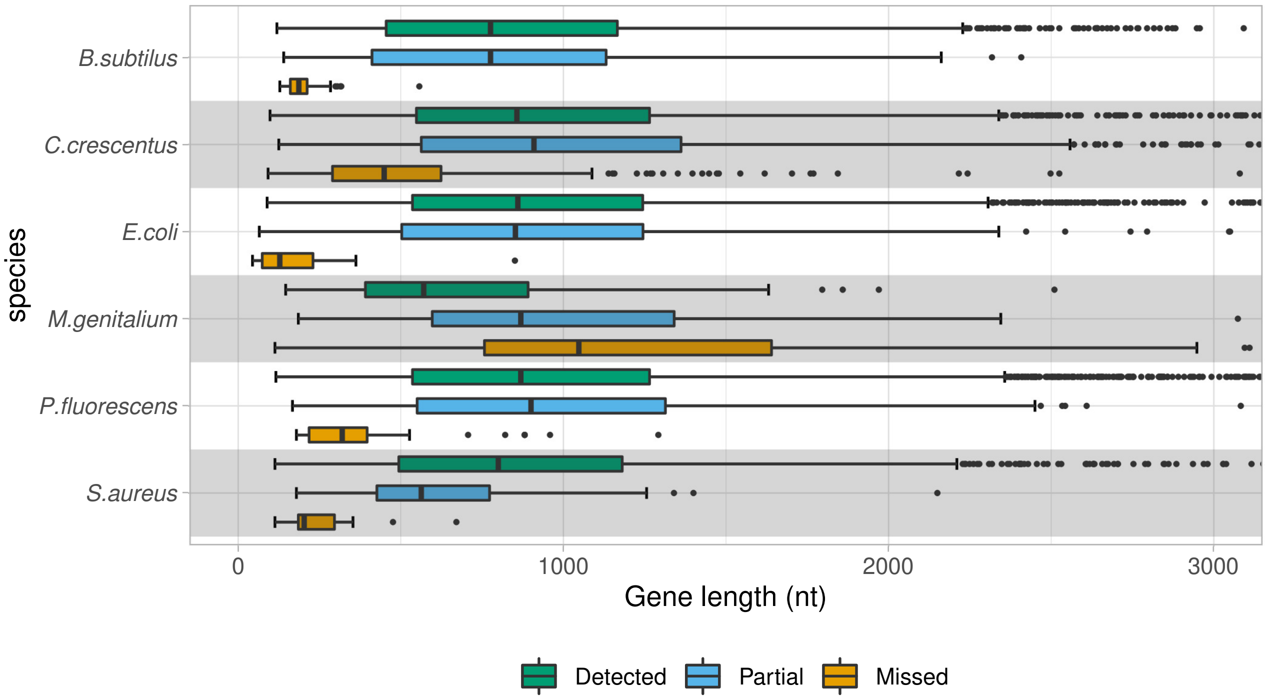

Figure 4: Lengths of Ensembl annotated CoDing Sequence (CDS) genes, those which were partially detected by Prodigal and those which were missed, for each model organism. The x-axis is truncated at 3,000 nt. With the exception of *M. genitalium*, the distributions of lengths of the missed genes are generally to the left of the distributions of the detected genes. Thus short genes are commonly overlooked by Prodigal and other tools.

3 Prediction Tools

3.1 Prediction Tools Run-Parameters

All tools were provided with the same 6 DNA data files each containing the complete genome for an organism in a single sequence. We did not provide genome-specific parameters such as alternative codon tables to the tools as this study aimed to be representative of real-world analysis where such information may not be known. Each tool was run using its default parameters with no user-defined filtering.

This was performed locally on a 64-bit Linux machine with an i7 2600k CPU with 32GB RAM, however none of the tools required more than a few minutes to run or more than 500 MB of RAM.

While most of the tools were available as online resources, they were downloaded from the links included in their associated publications and the specific versions used are listed below. Where no version number is available, the year of when the tool was used is listed.

3.2 Prediction Tools

- Model-based group:

  Some tools have been designed for a specific set genomes or strains and require a pre-built model (a rigid set of parameters tuned to a particular organism) to perform predictions. The construction of these models rely heavily on having an accurate and complete set of genes for a particular organism (among other information). While inaccuracies or biases in the data are likely to be present in the final models, model-based gene predictors trained on a particular species are expected to perform well on strains with comparable gene and genome structure. Overfitting can occur, where only similar genes to those previously found are detected at a high sensitivity. However, there can be large differences in gene number, gene length and genome size between strains of the same species. Model-based prediction for certain model organisms where specific strains are often used for scientific and industrial purposes can still be effective as there may be little genetic difference between two isolates of the same strain.

  The model-based tools were provided with two different organism models, *E. coli* K-12 and *S. aureus* - Mu50 (strains selected where possible). These were chosen as both were in the set of six bacteria and were models which were already available for all model-based tools. In addition, Augustus, which was originally developed for eukaryotic gene prediction, was run with the inclusion of the *H. sapiens* model and each individual Coding Sequence (CDS) predicted was retained as an independent predicted CDS.

  - **Augustus** Keller *et al.* (2011) - Version 3.3.3
    Originally published in 2003, Augustus was developed as a eukaryote genome prediction tool combining protein-family-based gene prediction and incorporated knowledge from external sources (pre-computed genome models) to combine them with an *ab initio* prediction to specifically help with exon prediction. Later versions of Augustus included 3 bacterial and 1 archaeal species to the pre-computed model list to allow for a selection of prokaryotic genome annotation.
  - **EasyGene** Nielsen and Krogh (2005) - Version 1.2
    EasyGene 1.2 published in 2005, employs a genome specific Hidden Markov Model (HMM) which after extracting all CDSs above 120 nt, filters them by using a sequence similarity search to a protein database. The resulting genes and their start positions are then used to retrain the HMM. EasyGene produces scores for multiple potential start codons for each gene and selects the one with the highest computed confidence value.
  - **GeneMark.hmm** Lukashin and Borodovsky (1998) - Prokaryote Model Version 3.2.5
    GeneMark.hmm, published in 1998 was developed to be one of the first tools to "improve the gene prediction quality in terms of finding exact gene boundaries". A HMM is used to model gene boundaries as transitions between hidden states along with ribosomal binding site patterns to refine translation initiation codons. The current genome model parameters were derived from the use of GeneMarkS, the successor of GeneMark.hmm.
  - **GeneMark** Borodovsky and McIninch (1993) - Version 2.5
    GeneMark, developed in 1993, was one of the first gene prediction methods to efficiently perform whole-genome annotation, notably for its ability to predict CDSs on both strands of DNA simultaneously. Markedly, GeneMark was used for the first annotation of a completely sequenced bacterium,

Haemophilus influenzae, and the first completely sequenced archaeon, *Methanococcus jannaschii.* The GeneMark algorithm consists of species-specific inhomogeneous Markov chain models computed from protein-coding DNA sequences and homogeneous Markov chain models of non-coding DNA. Probability of a predicted sequence fragment to be protein coding in one of six possible frames (including three frames in complementary DNA strand) or to be "non-coding" is computed to determine potential genes in the opposite strand of DNA.

  - **FGENESB** Salamov and Solovyevand (2011) - '2020' The FGENESB pipeline identifies protein, tRNA and rRNA genes, potential promoters, terminators and operons and performs an initial prediction of 'long' CDSs as a starting point for calculating parameters for gene prediction. The gene prediction algorithm is based on Markov chain models of coding regions and their translation and termination sites. Furthermore, operon prediction is performed using distances between CDSs, frequencies of neighboring genes in known bacterial genomes and positions of predicted promoters and terminators. FGENESB, unlike other model-based prediction tools, presents its model selection as "Choose closest organism", rather than "select species/organism", indicating that the developers acknowledge the models may be used as best-fit rather than for exact species prediction.

- *Ab initio* group:

  Self-training tools do not require any previous knowledge of the target genome and predict *ab initio*, directly from sequence. These were developed to be used on different prokaryotic organisms, however, they do rely on broad models either trained on features gathered directly from the input genome or predict CDSs using a set of predefined parameters which may be adapted. The criteria considered while making predictions include but are not limited to, overlapping CDSs, GC content, CDS length, predicted start and stop codons, and distances between CDSs Delcher *et al.* (1999); Besemer *et al.* (2001). Unfortunately, these criteria and their thresholds are still based on prior knowledge as deciding between candidate CDSs still requires a number of assumptions based on previously studied genes and genomes which the developer has embedded into the algorithm.

  Transdecoder, while technically an *ab initio* tool, is unlike the others in this group as it was specifically designed to predict CDS regions in transcript data.

  - **Prodigal** Hyatt *et al.* (2010) - Version 2.6.2 Prodigal is an unsupervised gene predictor which examines the input genome for the creation of its input-specific training set. 100 prokaryote genomes were selected in the initial development of the algorithm to determine "very general rules about the nature of prokaryotic genes, such as gene size, maximum overlap between two genes... and RBS (ribosomal binding site) motif usage". A number of constants within the algorithm were tuned to the genetic makeup of the 100 genomes. GC is an important statistic for Prodigal and it is used for a number of steps in the prediction process such as coding scores for each gene predicted. Prodigal performs a number of scoring functions on different aspects of each DNA region selected, thus producing a set of putative "most-likely real" genes. These genes are then examined and are used to tune the model before prediction of genes which exhibit lower likelihood scores. Furthermore, Prodigal has been designed to detect whether genetic code 4 is needed (*Mysoplasma*) and use it instead of the default code 11.
  - **GeneMarkS** Besemer *et al.* (2001) - Version 4.25 Developed in 2001, GeneMarkS was one of the first *ab initio* gene prediction methods which could learn directly from short (>400) sequences without prior knowledge or pre-trained models. As with other contemporary tools, HMMs were trained on protein-coding sequence data, non-coding DNA samples and modelled on transition and initiation parameters trained from input sequence. Codon frequencies and positional statistics are utilised along with genomic GC content to learn coding potential for identified CDSs. GeneMarkS has become a bedrock for future prediction tools and has been used as part of wider genome annotation pipelines.
  - **GeneMarkS 2** Lomsadze *et al.* (2018) - Version '2020' An advancement over the original GeneMarkS tool, GeneMarkS-2 further utilises a self-derived *ab initio* training model learnt from input sequences for finding species-specific (native) genes. A collection of pre-computed "heuristic" models are utilised to identify harder-to-detect genes (horizontally transferred). GeneMarkS-2 learns distinct sequence patterns inherent to prokaryotic genomes which are involved in gene expression control. The majority of protein-coding regions in prokaryotic genomes are known to carry species-specific codon usage

patterns and GeneMarkS-2 learns these patterns and estimates parameters of typical protein-coding regions of a target genome. This process is similar to the one employed by GeneMarkS(1) but extended.

- **GLIMMER 3** Delcher *et al.* (2007) - Version 3.02 GLIMMER 3, published in 2007 is the third iteration of the GLIMMER microbial gene predictor software. A number of improvements over the previous implementations include improved coding region and start codon detection, along with a reduction in incorrectly reported overlapping genes. GLIMMER 3, as with the previous versions, starts by predicting CDSs with little filtering and then using a number of user defined (or default) parameters (such as start codon selection, CDS length and overlap length). These CDSs are then scored for their coding potential. To overcome the high levels of potential false positive overlapping CDSs, GLIMMER3 uses these scores to select which of any two overlapping CDSs are more likely to be real (in cases where maximum overlap is surpassed). An Interpolated Markov Model (IMM) is used in the prediction process to help identify coding regions and has also been shown to separate DNA between bacterium and host DNA. GLIMMER, along with GeneMarkS, was also used as part of the NCBI prokaryote annotation pipeline (Tatusova *et al.*, 2016).

- **GeneMark Heuristic Approach** Besemer and Borodovsky (1999) - Version 3.25 As with many other tools from the GeneMark suite, GeneMark Heuristic Approach (GeneMark HA) was developed on the observations made from GeneMark and GeneMark.hmm. The method was designed to build Markov models derived on a minimal amount of DNA information from 17 completed bacterial genomes. Linear regression was performed to approximate relationships between positional and global nucleotide frequencies, relationships between the amino acid frequencies and the global GC% of the bacterial genomes. Amino acids frequencies were calculated mostly from an *E. coli* genome to build constants for the algorithm. The algorithm builds a heuristic model for every sequence longer than 400 nt. GeneMark HA derived models are expected to be applied to the analysis of the input sequence by the GeneMark and GeneMark.hmm programs.

- **TransDecoder** Haas *et al.* (2013) - 5.5.0 TransDecoder was designed to identify candidate coding regions within transcript sequences, such as those generated by *de novo* RNA-Seq transcript assembly, or constructed based on RNA-Seq alignments to the genome. TransDecoder identifies likely coding sequences based on a minimum ORF length and a computed log-likelihood score >0. The coding score is greatest when the ORF is scored in the 1st reading frame as compared to scores in the other 5 reading frames. The longer of two CDSs is reported if one is encapsulated by the others coordinates. However, a single transcript can report multiple CDSs (allowing for operons, chimeras, etc).

- **FragGeneScan** Rho *et al.* (2010) - 1.3.0 FragGeneScan has been specifically designed to improve prediction performance on metagenomic and short-read sequence data with high levels of sequencing errors, but also perform comparably with other contemporary tools on complete genomes. A combination of probabilistic models trained on codon usage and sequence error data, was used to evaluate sections of DNA for their gene encoding potential. This method has shown higher performance for predicting genes on short-reads with high levels of sequence error than other contemporary methods but can be used on complete low-error genomes.

- **MetaGene** Noguchi *et al.* (2006) - 2.24.0 MetaGene, one of the first gene prediction tools specifically developed for prediction on fragmented and metagenomic genomes, examines di-codon frequencies estimated by the GC content of a given sequence with other measures such as length, distance between CDSs and start codon distribution. MetaGene can predict a whole range of prokaryotic genes based on the anonymous genomic sequences of a few hundred bases and identify partial CDSs which have are located on the terminus of the fragmentary genomic sequences.

- **MetaGeneMark** Zhu *et al.* (2010) - '2020' The heuristic model behind MetaGeneMark was developed to replace traditional methods of ORF prediction parameter estimation such as supervised training on a set of "validated" genes or unsupervised training on an input sequence. Dependencies which had formed in evolution, between codon frequencies and genome nucleotide composition are utilised to derive patterns of codon frequencies, critical for the model parameterisation, from frequencies of nucleotides observed in a short or metagenomic sequences. An effective method to estimate prediction parameters was derived from the frequencies of oligonucleotides in protein-coding regions and whole-genome nucleotide composition.

– **Meta Gene Annotator** Noguchi *et al.* (2008) - Version '2008/8/19' Published in 2008, MetaGeneAnnotator predicts all kinds of prokaryotic genes from anonymous genomic sequences. It integrates statistical models of prophage, bacterial and archaeal genes, and builds a self-trained model from input sequences for the predictions. This results in the detection of not only "typical genes but also atypical genes, such as horizontally transferred and prophage genes in a prokaryotic genome". The algorithm also includes a novel approach for the analysis of ribosomal binding sites, which has enabled the detection of species-specific patterns, thus allowing for "precise" prediction of translation starts sites.

Metagenomic gene predictors form a subset of *ab initio* self-training tools which primarily rely on the same methods but involve additional parameters. They must contend with a number of additional difficulties common to metagenomic annotation. The dynamics of metagenomic DNA sequences such as chimeric contigs assembled from different organisms, cause a number of problems for even self-learning predictors. Any model or parameters chosen would need to be recalculated for every metagenomic contig as each is likely to be from a different organism and therefore have different characteristics. Metagenomic assemblies often consist of fragmented genomes which can lead to a number of problems for gene prediction. A given contig may only contain a fragment of a gene. Therefore, simply looking for start and stop codons, which may not be present, along with changes in GC content outside of predicted gene regions, will not be as useful to help to distinguish between coding and non-coding regions. These errors are extremely difficult to account for and tools have been produced to tackle them directly Rho *et al.* (2010) We have included 3 metagenome prediction tools in this study.

Many of the tools comprise different versions of the same core software but produced differing results. For example, GeneMark, MetaGeneMark, GeneMark Heuristic Approach, GeneMark Hidden Markov Model, GeneMark S and GeneMark S2 were all from the same suite of tools and have many similarities with each other but are designed for different purposes and produce different results.

It was decided that no specific rules were to be enforced on the tools. Each tool was run using its default parameters and this was to get a baseline for their accuracy with the least amount of human support. Many hard-coded assumptions were consistent across the tools, such as minimum ORF length and the codons allowed to identify the start and end of an ORF. Some of the tools allowed the minimum ORF length to be altered, but the majority fixed the threshold to around 100 nucleotides.

4 Code and analysis scripts

To inspect the various differences between the six genomes, a number of additional Python3 scripts were written to interrogate the canonical annotations. These scripts are available at `https://github.com/NickJD/ORForise/tree/master/src/ORForise/ORForise_Analysis`).

Python3 (Van Rossum and Drake, 2009) with Matplotlib (Hunter, 2007) and R (R Core Team, 2020) with ggplot2 (Wickham, 2016) were used to produce the figures.

5 Description of Comparison Metrics

- Number of Predicted CDSs:
This is the number of CDSs that the tool has predicted. Some tools predict a large number of potential CDSs and then filter them. All 'Predicted CDS' metrics correspond to the remaining predicted CDSs presented to the user after default filtering.

- Percentage Difference of Number of Predicted CDSs: **(M3)**
This is the percentage change between the number of predicted CDSs and the number of actual reference Genes. *100 * (Number of predicted CDSs - Number of reference Genes) / Number of reference Genes*

- Number of Predicted CDSs that Detect a Gene:
This is the number of CDSs that correctly detect at least 75% of the nucleotides of a reference Gene and are in the same frame.

- Percentage of Predicted CDSs that Detected a Gene: **(M2)**
This is the percentage of predicted CDSs that correctly detect at least 75% of the nucleotides of a reference Gene and are in the same frame.

- Number of Genes Detected:
The number of reference Genes Detected is characterised as the number of predicted CDS which are in frame with a reference gene and has captured at least 75% of its nucleotide sequence.

- Percentage of Genes Detected: **(M1)**
The percentage of reference Genes Detected is characterised as the percentage of predicted CDS which are in frame with a reference gene and has captured at least 75% of its nucleotide sequence.

- Median Length of All Predicted CDSs:
Median length of all predicted CDSs, in nucleotides.

- Percentage Difference of Median CDS Length: **(M4)**
This is the Percentage Difference from the mean length of reference Genes compared to the mean length of all predicted CDSs. *100 * (Median CDS length - reference gene median length) / reference gene median length*

- Minimum Length of All Predicted CDSs:
The length of the shortest predicted CDS, in nucleotides.

- Minimum Length Difference:
This is the percentage difference from the shortest reference gene compared to the length of the shortest predicted CDS. *100 * (Minimum CDS length - Minimum reference gene length) / Minimum reference gene length*

- Maximum Length of All Predicted CDSs:
The length of the longest predicted CDS, in nucleotides.

- Maximum Length Difference:
This is the percentage difference from the longest reference Gene compared to the length of the longest predicted CDS. *100 * (Maximum CDS length - Maximum reference gene length) / Maximum reference gene length*

- Median GC Content of All Predicted CDSs:
This median GC content calculated from all predicted CDSs.

- Percentage Difference of All Predicted CDSs Median GC:
This is the Percentage Difference of the median GC content of all predicted CDSs compared to the median GC content of all reference Genes. *100 * (Median GC content of all CDSs - Median GC content of all reference genes) / Median GC content of all reference genes*

- Median GC Content of Matched Predicted CDSs:
This median GC content calculated from predicted CDSs that detected a reference gene.

- Percentage Difference of Matched Predicted CDS GC:
  This is the Percentage Difference of the median GC content of predicted CDSs that detected a reference gene compared to the median GC content of all reference genes. *100 * (Median GC content of matched CDSs - Median GC content of all reference genes) / Median GC content of all reference genes*

- Number of Predicted CDSs that Overlap Another Predicted CDS:
  This is the number of predicted CDSs that overlap another predicted CDS by at least one nucleotide base.

- Percentage Difference of Overlapping Predicted CDSs:
  This is the Percentage Difference of overlapping predicted CDSs as compared to the number of overlapping reference Genes. *100 * (Number of overlapping CDSs - Number of overlapping reference genes) / Number of overlapping reference genes*

- Maximum Length of Predicted CDS Overlap:
  This is the maximum length of predicted CDS overlap, in nucleotides.

- Median Length of Predicted CDS Overlap:
  This is the median length of predicted CDS overlap calculated from all CDS overlap lengths.

- Number of Matched Predicted CDSs Overlapping Another Predicted CDS:
  This is the number of predicted CDSs that detected a reference gene that overlap another predicted CDS by at least one base.

- Percentage Difference of Matched Overlapping Predicted CDSs: **(M8)**
  This is the percentage difference of overlapping CDSs that detected a reference gene as compared to the number of overlapping annotated reference genes. *100 * (Number of matched overlapping CDSs - Number of overlapping reference genes) / Number of overlapping reference genes*

- Maximum Length of Matched Predicted CDS Overlap:
  This is the maximum length of matched predicted CDS overlap, in nucleotides.

- Median Length of Matched Predicted CDS Overlap:
  This is the median length of matched predicted CDS overlap calculated from all predicted CDS overlap lengths.

- Number of Short Predicted CDSs:
  This is the number of predicted CDSs that are under 100 nucleotide bases.

- Percentage Difference of Short Predicted CDSs:
  This is the percentage difference of predicted short CDSs as compared to the number of annotated reference short genes (short defined as less than 100 nucleotide bases). *100 * (Number of short CDSs - Number of short genes) / Number of short genes*

- Number of Matched Short Predicted CDSs:
  This is the number of CDSs which detected a reference gene and that are under 100 nucleotide bases.

- Percentage Difference of Matched Short Predicted CDSs: **(M9)**
  This is the percentage difference of short CDSs which detected a gene as compared to the number of reference short genes. *100 * (Number of s short matched CDSs - Number of short genes) / Number of short genes*

- Number of Perfect Matches: **(M5)**
  This is the number of predicted CDSs that have correctly identified the exact start and stop position of a reference gene.

- Percentage of Perfect Matches:
  This is the percentage of CDSs that have correctly identified the exact start and stop position of a reference gene. *100 * Number of CDSs which matched a reference gene - Number of reference genes) / Number of reference genes*

- Number of Perfect Starts:
  This is the number of Matched CDSs that have correctly identified the start position of a reference gene.
- Percentage of Perfect Starts:
  This is the percentage of Matched predicted CDSs that have correctly identified a reference gene and its start position.
- Number of Perfect Stops:
  This is the number of matched predicted CDSs that have correctly identified the stop position of a reference gene.
- Percentage of Perfect Stops:
  This is the percentage of matched CDSs that have correctly identified a reference gene and its stop position.
- Number of Out of Frame Predicted CDSs:
  This is the number of predicted CDSs that covered more than 75% of a reference gene but were out of frame, thus classified as Unmatched.
- Number of Matched Predicted CDSs Extending a Coding Region:
  This is the number of matched predicted CDSs that extend the 3 and 5-prime end of its detected reference gene.
- Percentage of Matched Predicted CDSs Extending a Coding Region:
  This is the percentage of matched CDSs that extend the 3 and 5-prime end of its detected reference gene.
- Number of Matched Predicted CDSs Extending Start Region:
  This is the number of matched predicted CDSs that extend the 5-prime end of its detected reference gene.
- Percentage of Matched Predicted CDSs Extending Start Region:
  This is the percentage of matched CDSs that extend the 5-prime end of its detected reference gene.
- Number of Matched Predicted CDSs Extending Stop Region:
  This is the number of matched CDSs that extend the 3-prime end of its detected reference gene.
- Percentage of Matched Predicted CDSs Extending Stop Region:
  This is the percentage of matched CDSs that extend the 3-prime end of its detected reference gene.
- Number of All Predicted CDSs on Positive Strand:
  This is the number of all predicted CDSs on the positive strand.
- Percentage of All Predicted CDSs in Positive Strand:
  This is the percentage of all predicted CDSs on the positive strand.
- Number of All Predicted CDSs in Negative Strand:
  This is the number of all predicted CDSs on the negative strand.
- Percentage of All Predicted CDSs in Negative Strand:
  This is the percentage of all predicted CDSs on the negative strand.
- Median Start Difference of Matched Predicted CDSs: **(M6)**:
  This is the median difference calculated by taking all matched predicted CDSs start position differences from the detected reference genes and finding the median of these differences. This is calculated in nucleotides and the closer to 0, the lower the difference or effective error.
- Median Stop Difference of Matched Predicted CDSs: **(M7)**
  This is the median difference calculated by taking all matched predicted CDSs stop position differences from the detected reference genes and finding the median of these differences. This is calculated in nucleotides and the closer to 0, the lower the difference or effective error.
- ATG Start Percentage:
  This is the percentage of all predicted CDSs which begin with the ATG codon.

- GTG Start Percentage:
  This is the percentage of all predicted CDSs which begin with the GTG codon.
- TTG Start Percentage:
  This is the percentage of all predicted CDSs which begin with the TTG codon.
- ATT Start Percentage:
  This is the percentage of all predicted CDSs which begin with the ATT codon.
- CTG Start Percentage:
  This is the percentage of all predicted CDSs which begin with the CTG codon.
- Other Start Codon Percentage:
  This is the percentage of all predicted CDSs which begin with an alternative start codon.
- TAG Stop Percentage:
  This is the percentage of all predicted CDSs which end with the TAG codon.
- TAA Stop Percentage:
  This is the percentage of all predicted CDSs which end with the TAA codon.
- TGA Stop Percentage:
  This is the percentage of all predicted CDSs which end with the TGA codon.
- Other Stop Codon Percentage:
  This is the percentage of all predicted CDSs which end with an alternative stop codon.
- True Positive:
  The true positive value is calculated by dividing the number of reference genes correctly detected by the total number of reference genes (75% detected and in frame). *Number of reference CDSs detected / Number of reference genes*
- False Positive:
  The false positive value is calculated by dividing the number of predicted CDSs which did not match any reference genes by the total number of reference genes.*Number of unmatched CDSs / Number of reference genes*
- False Negative:
  The false negative value is calculated by dividing the number of reference genes missed by the predicted CDSs by the total number of reference genes.
- Precision: **(M10)**
  The precision value is calculated by dividing the true positive value by the sum of the true positive and false positive values.
- Recall: **(M11)**
  The recall value is calculated by dividing the true positive value by the sum of the true positive and false negative values.
- False Discovery Rate: **(M12)**
  The false discovery rate is calculated by dividing the false positive value by the sum of the false positive and true positive values.
- True Positive (Nucleotide):
  The true positive value is calculated by dividing the number of nucleotides in reference genes correctly detected by the total number of nucleotides in all reference genes.
- False Positive (Nucleotide):
  The false positive value is calculated by dividing the number of nucleotides in predicted CDSs but not in any reference genes by the total number of nucleotides not in any reference genes.

- True Negative (Nucleotide):
  The true negative value is calculated by dividing the number of nucleotides not in any predicted CDSs by the number of nucleotides not in any reference genes.

- False Negative (Nucleotide):
  The false negative value is calculated by dividing the number of nucleotides in reference genes but not in predicted CDSs by the total number of nucleotides in all reference genes.

- Precision (Nucleotide):
  This precision value is calculated by dividing the nucleotide true positive value by the sum of the nucleotide true positive and false positive values.

- Recall (Nucleotide):
  This recall value is calculated by dividing the nucleotide true positive value by the sum of the nucleotide true positive and false negative values.

- False Discovery Rate (Nucleotide):
  This false discovery rate is calculated by dividing the nucleotide false positive value by the sum of the nucleotide false positive and true positive values.

- Predicted CDS Nucleotide Coverage of Genome:
  This is the percentage of nucleotides in all predicted CDSs out of all nucleotides in the genome.

- Correctly Matched CDS Nucleotide Coverage of Genome:
  This is the percentage of nucleotides in Matched CDSs which correctly detected a reference gene out of all nucleotides in the genome.

6 Supplementary Tables

Table 1: Start codon usage for Current Ensembl Annotation CoDing Sequence (CDS) genes for the six model organisms. Note the variation in usage of canonical start codon ATG and the alternative GTG and TTG codons.

Model Organism	ATG	GTG	TTG	ATT	CTG	Other
B. subtilis	76.81%	10.10%	13.09%	0.00%	0.00%	0.00%
C. crescentus	68.58%	17.69%	13.73%	0.00%	0.00%	0.00%
E. coli	90.67%	7.50%	1.70%	0.05%	0.05%	0.02%
M. genitalium	88.45%	7.56%	3.99%	0.00%	0.00%	0.00%
P. fluorescens	88.55%	7.55%	2.92%	0.21%	0.48%	0.29%
S. aureus	86.80%	6.62%	6.58%	0.00%	0.00%	0.00%

Table 2: Start codon substitution table for genes which were misreported on the 5' prime end by Prodigal, combined for the six model organisms. Column headers represent Ensembl annotated start codons and row headers represent the incorrectly predicted start codons, having chosen an alternative further upstream or downstream of the true start codon. The last row, 'Correct codon', shows the numbers of Perfect Match predicted CDSs by Prodigal with the specified start codons. Further start codons with low usage were combined into the category labelled 'other'.

	Correct codon				
	ATG	GTG	TTG	CTG	Other
Incorrect ATG	817	371	357	19	24
Incorrect GTG	106	76	49	4	0
Incorrect TTG	81	47	37	3	3
Incorrect CTG	0	0	0	0	1
Incorrect other	0	0	0	0	0
Correct codon	14933	1321	847	0	0

Table 3: Stop codon usage for Current Ensembl Annotation CoDing Sequence (CDS) genes for the six model organisms. *M. genitalium* recodes TGA for Tryptophan and *E. coli* uses CTT for one gene.

Model Organism	TAG	TAA	TGA	Other
B. subtilis	13.96%	62.93%	23.11%	0.00%
C. crescentus	32.78%	19.86%	47.36%	0.00%
E. coli	6.89%	64.68%	28.41%	0.02%
M. genitalium	27.10%	72.90%	0.00%	0.00%
P. fluorescens	14.18%	30.42%	55.41%	0.00%
S. aureus	15.01%	74.17%	10.82%	0.00%

Table 4: GC content differences for Prodigal annotations. Shown here as median values are: GC content of Current Ensembl Annotation CoDing Sequence (CDS) genes, the genes detected by Prodigal, those Prodigal obtained a partial match and those it missed.

Model Organism	Ensembl GC	Detected GC	Partial GC	Missed GC
B. subtilis	44.19%	44.25%	43.99%	39.13%
C. crescentus	67.52%	67.71%	67.69%	65.65%
E. coli	52.15%	52.21%	52.14%	43.14%
M. genitalium	31.44%	32.90%	32.75%	30.76%
P. fluorescens	61.25%	61.36%	60.25%	53.36%
S. aureus	33.33%	33.33%	30.13%	32.62%

Table 5: Percentages of the Current Ensembl Annotation CoDing Sequence (CDS) genes and Predicted CDSs identified as overlapping. We show averages for *ab initio* and model-based predicted CDSs.

Model Organism	Ensembl	*Ab initio*	Model-Based
B. subtilis	21.37%	21.44%	15.44%
C. crescentus	32.73%	25.51%	21.84%
E. coli	22.53%	22.68%	18.20%
M. genitalium	46.43%	16.47%	11.65%
P. fluorescens	24.16%	25.42%	18.08%
S. aureus	19.61%	19.98%	15.72%

Table 6: Percentage Difference of overlapping predicted CDSs as compared to the Current Ensembl Annotation CoDing Sequence (CDS) genes. *Ab initio* and model based tools are separated into 2 groups each. 'Matched' represents the Percentage Difference for those predicted CDSs which were able to detect an Current Ensembl Annotation CDS gene whereas 'All' represents the Percentage Difference of the number of overlapping predicted CDSs across all predicted CDSs.

Group	Average	Standard Deviation	Standard Error
Matched, *ab initio*	-23.62%	7.16%	2.27%
Matched, model	-52.89%	24.79%	7.47%
All, *ab initio*	-6.07%	11.55%	3.65%
All, model	-30.15%	29.41%	8.87%

Table 7: Percentage of the Current Ensembl Annotation CoDing Sequence (CDS) genes and predicted CDSs categorised as Short CDSs (≤100 amino acids). We show averages for *ab initio* and model-based predicted CDSs. Note the large increase in Short CDSs predicted for *M. genitalium*.

Model Organism	Ensembl	*Ab initio*	Model-based
B. subtilis	13.66%	12.58%	13.24%
C. crescentus	7.60%	8.11%	15.33%
E. coli	10.24%	10.45%	13.04%
M. genitalium	4.83%	38.44%	36.99%
P. fluorescens	7.84%	9.06%	19.01%
S. aureus	10.05%	11.26%	15.59%

Table 8: Percentage Difference of short predicted CDSs (≤100 amino acids) as compared to the Current Ensembl Annotation CDS genes. *Ab initio* and model based tools are separated into 2 groups each. 'Matched' represents the Percentage Difference for those predicted CDSs which were able to detect a Current Ensembl Annotation CDS gene whereas 'All' represents the Percentage Difference of the number of Short CDSs across all predicted CDSs. The results from *M. genitalium* were not included in this table's calculations.

Group	Average	Standard Deviation	Standard Error
Matched, *ab initio*	-26.38%	25.68%	8.12%
Matched, model	-53.69%	21.71%	6.55%
All, *ab initio*	9.07%	39.87%	12.61%
All, model	39.10%	91.22%	27.50%

Table 9: *M. genitalium*-only Percentage Difference of short CDSs (≤100 amino acids) as compared to the Current Ensembl Annotation CoDing Sequence (CDS) genes. *Ab initio* and model based tools are separated into 2 groups each. 'Matched' represents the Percentage Difference for those CDSs which were able to detect a Current Ensembl Annotation CDS gene whereas 'All' represents the Percentage Difference of the number of Short CDSs across all predicted CDSs.

Group	Average	Standard Deviation	Standard Error
Matched, *ab initio*	-27.34%	25.15%	7.95%
Matched, model	-55.28%	20.62%	6.22%
All, *ab initio*	261.11%	139.28%	44.04%
All, model	148.00%	164.58%	49.62%

Table 10: Aggregated tool predictions provide a small increase in Percentage of Genes Detected (M1) but over-predict a large number of additional CDSs. Here we compare the 'best tool' (tool with highest M1 score) predictions versus 'aggregated tools' (combination of predictions from top 5 ranked tools; Prodigal, GeneMark-S-2, MetaGeneAnnotator, MetaGeneMark and GeneMark-S) for the percentage of detected genes, partial matches and additional over-predictions made by the aggregated tools which did not detect a Current Ensembl Annotation CDS gene. GeneMark.hmm results are reported for *S. aureus* as even though it performed joint best with GeneMarkS (M1), it reported a higher proportion of Perfect Matches (M5). Agg' and Perc' stand for Aggredate and Percentage respectively

Model Organism	CEA CDS	Best Tool	Best Tool Detected **[Partial Matches]**	Agg' Detected **[Partial Matches]**	Best Tool CDSs	Agg' Extra CDSs **[Per' Increase]**
B. subtilis	4,011	MetaGeneAnnotator	99.85% [**1.40%**]	100% [**0.37%**]	4,058	1,692 [**41.09%**]
C. crescentus	3,737	MetaGeneMark	92.83% [**31.62%**]	93.66% [**23.17%**]	3,770	1,304 [**34.59%**]
E. coli	4,052	Prodigal	98.05% [**5.94%**]	98.82% [**1.57%**]	4,253	1,635 [**38.44%**]
M. genitalium	476	Prodigal	39.92% [**32.63%**]	40.13% [**30.89%**]	995	426 [**42.81%**]
P. fluorescens	5,178	GeneMarkS	99.29% [**12.97%**]	99.92% [**3.05%**]	5,513	1,891 [**34.03%**]
S. aureus	2,478	GeneMark.hmm (*S. aureus* model)	99.60% [**4.58%**]	99.84% [**0.28%**]	2,582	774 [**29.98%**]

Table 11: Numbers of additional CDSs predicted by Prodigal that can be added to Ensembl gene annotations. Additional CDSs are chosen if there are no fewer than 50 nucleotides overlapping with an Ensembl gene.

Model Organism	Ensembl Genes	Additional Prodigal CDSs
B. subtilis	4,011	62
C. crescentus	3,737	64
E. coli	4,052	270
M. genitalium	476	70
P. fluorescens	5,178	293
S. aureus	2,478	74

Table 12: Numbers of Ensembl genes which form an intersection (100% or 75%) with CDSs predicted by Prodigal.

Model Organism	Ensembl Genes	Prodigal CDSs	100% Intersection	75% Intersection
B. subtilis	4,011	4,016	3,673	3,943
C. crescentus	3,737	3,704	2,393	3,433
E. coli	4,052	4,263	3,737	3,973
M. genitalium	476	995	128	190
P. fluorescens	5,178	5,421	4,736	5,100
S. aureus	2,478	2,534	2,434	2,457

References

Anton, B. P., Mongodin, E. F., Agrawal, S., Fomenkov, A., Byrd, D. R., Roberts, R. J., and Raleigh, E. A. (2015). Complete genome sequence of ER2796, a DNA methyltransferase-deficient strain of *Escherichia coli* K-12. *PloS One*, **10**(5), e0127446.

Besemer, J. and Borodovsky, M. (1999). Heuristic approach to deriving models for gene finding. *Nucleic Acids Research*, **27**(19), 3911–3920.

Besemer, J., Lomsadze, A., and Borodovsky, M. (2001). GeneMarkS: a self-training method for prediction of gene starts in microbial genomes. Implications for finding sequence motifs in regulatory regions. *Nucleic Acids Research*, **29**(12), 2607–2618.

Borodovsky, M. and McIninch, J. (1993). GENMARK: Parallel gene recognition for both DNA strands. *Computers & Chemistry*, **17**(2), 123–133.

Delcher, A. L., Harmon, D., Kasif, S., White, O., and Salzberg, S. L. (1999). Improved microbial gene identification with GLIMMER. *Nucleic Acids Research*, **27**(23), 4636–4641.

Delcher, A. L., Bratke, K. A., Powers, E. C., and Salzberg, S. L. (2007). Identifying bacterial genes and endosymbiont DNA with Glimmer. *Bioinformatics*, **23**(6), 673–679.

Dueholm, M. S., Danielsen, H. N., and Nielsen, P. H. (2014). Complete genome sequence of *Pseudomonas sp. UK4*, a model organism for studies of functional amyloids in *pseudomonas*. *Genome Announc.*, **2**(5), e00898–14.

Glass, J. I., Assad-Garcia, N., Alperovich, N., Yooseph, S., Lewis, M. R., Maruf, M., Hutchison, C. A., Smith, H. O., and Venter, J. C. (2006). Essential genes of a minimal bacterium. *Proceedings of the National Academy of Sciences*, **103**(2), 425–430.

Haas, B. J., Papanicolaou, A., Yassour, M., *et al.* (2013). *De novo* transcript sequence reconstruction from RNA-seq using the Trinity platform for reference generation and analysis. *Nature Protocols*, **8**(8), 1494–1512.

Hunter, J. D. (2007). Matplotlib: A 2d graphics environment. *IEEE Annals of the History of Computing*, **9**(03), 90–95.

Hutchison, C. A., Peterson, S. N., Gill, S. R., Cline, R. T., White, O., Fraser, C. M., Smith, H. O., and Venter, J. C. (1999). Global transposon mutagenesis and a minimal *Mycoplasma* genome. *Science*, **286**(5447), 2165–2169.

Hyatt, D., Chen, G.-L., LoCascio, P. F., Land, M. L., Larimer, F. W., and Hauser, L. J. (2010). Prodigal: prokaryotic gene recognition and translation initiation site identification. *BMC Bioinformatics*, **11**(1), 119.

Itaya, M., Tsuge, K., Koizumi, M., and Fujita, K. (2005). Combining two genomes in one cell: stable cloning of the synechocystis pcc6803 genome in the *Bacillus subtilis* 168 genome. *Proceedings of the National Academy of Sciences*, **102**(44), 15971–15976.

Keller, O., Kollmar, M., Stanke, M., and Waack, S. (2011). A novel hybrid gene prediction method employing protein multiple sequence alignments. *Bioinformatics*, **27**(6), 757–763.

Lomsadze, A., Gemayel, K., Tang, S., and Borodovsky, M. (2018). Modeling leaderless transcription and atypical genes results in more accurate gene prediction in prokaryotes. *Genome Research*, **28**(7), 1079–1089.

Lukashin, A. V. and Borodovsky, M. (1998). GeneMark.hmm: new solutions for gene finding. *Nucleic Acids Research*, **26**(4), 1107–1115.

Nielsen, P. and Krogh, A. (2005). Large-scale prokaryotic gene prediction and comparison to genome annotation. *Bioinformatics*, **21**(24), 4322–4329.

Nierman, W. C., Feldblyum, T. V., Laub, M. T., Paulsen, I. T., Nelson, K. E., Eisen, J., Heidelberg, J. F., Alley, M., Ohta, N., Maddock, J. R., *et al.* (2001). Complete genome sequence of *Caulobacter crescentus*. *Proceedings of the National Academy of Sciences*, **98**(7), 4136–4141.

Noguchi, H., Park, J., and Takagi, T. (2006). MetaGene: prokaryotic gene finding from environmental genome shotgun sequences. *Nucleic Acids Research*, **34**(19), 5623–5630.

Noguchi, H., Taniguchi, T., and Itoh, T. (2008). MetaGeneAnnotator: detecting species-specific patterns of ribosomal binding site for precise gene prediction in anonymous prokaryotic and phage genomes. *DNA Research*, **15**(6), 387–396.

Parker, D., Narechania, A., Sebra, R., Deikus, G., LaRussa, S., Ryan, C., Smith, H., Prince, A., Mathema, B., Ratner, A. J., *et al.* (2014). Genome sequence of bacterial interference strain *Staphylococcus aureus 502A*. *Genome Announcements*, **2**(2), e00284–14.

R Core Team (2020). *R: A Language and Environment for Statistical Computing*. R Foundation for Statistical Computing, Vienna, Austria.

Rho, M., Tang, H., and Ye, Y. (2010). FragGeneScan: predicting genes in short and error-prone reads. *Nucleic Acids Research*, **38**(20), e191–e191.

Salamov, V. S. A. and Solovyevand, A. (2011). Automatic annotation of microbial genomes and metagenomic sequences. *Metagenomics and its applications in agriculture. Nova Science Publishers, Hauppauge*, pages 61–78.

Tatusova, T., DiCuccio, M., Badretdin, A., *et al.* (2016). NCBI prokaryotic genome annotation pipeline. *Nucleic Acids Research*, **44**(14), 6614–6624.

Van Rossum, G. and Drake, F. L. (2009). *Python 3 Reference Manual*. CreateSpace, Scotts Valley, CA.

Wickham, H. (2016). *ggplot2: Elegant Graphics for Data Analysis*. Springer-Verlag New York.

Zhu, W., Lomsadze, A., and Borodovsky, M. (2010). *Ab initio* gene identification in metagenomic sequences. *Nucleic Acids Research*, **38**(12), e132–e132.
